# IL-4-dependent Jagged1 expression/processing is associated with survival of chronic lymphocytic leukemia cells but not with Notch activation

**DOI:** 10.1038/s41419-018-1185-6

**Published:** 2018-11-26

**Authors:** Filomena De Falco, Beatrice Del Papa, Stefano Baldoni, Rita Sabatini, Franca Falzetti, Mauro Di Ianni, Maria Paola Martelli, Federica Mezzasoma, Maria Pelullo, Pierfrancesco Marconi, Paolo Sportoletti, Isabella Screpanti, Emanuela Rosati

**Affiliations:** 1 Department of Medicine, Institute of Hematology-Centro di Ricerche Emato-Oncologiche (CREO), University of Perugia, Perugia, Italy; 2 Department of Life, Health and Environmental Sciences, Hematology Section, University of L’Aquila, L’Aquila, Italy; 3 Department of Medicine and Aging Sciences, University of Chieti-Pescara, Chieti, Italy; 4 Department of Hematology, Transfusion Medicine and Biotechnologies, Ospedale Civile, Pescara, Italy; 5 Department of Molecular Medicine, University of Rome “La Sapienza”, Rome, Italy; 6 Department of Experimental Medicine, Biosciences and Medical Embryology Section, University of Perugia, Perugia, Italy

## Abstract

As previously reported, chronic lymphocytic leukemia (CLL) cells show constitutive Notch1/2 activation and express the Notchligand Jagged1. Despite increasing knowledge of the impact of Notch alterations on CLL biology and pathogenesis, the role of Jagged1 expressed in CLL cells remains undefined. In other cell types, it has been shown that after Notch engagement, Jagged1 not only activates Notch in signal-receiving cell, but also undergoes proteolytic activation in signal-sending cell, triggering a signaling with biological effects. We investigated whether Jagged1 expressed in CLL cells undergoes proteolytic processing and/or is able to induce Notch activation through autocrine/paracrine loops, focusing on the effect that CLL prosurvival factor IL-4 could exert on the Notch-Jagged1 system in these cells. We found that Jagged1 was constitutively processed in CLL cells and generated an intracellular fragment that translocated into the nucleus, and an extracellular fragment released into the culture supernatant. IL-4 enhanced expression of Jagged1 and its intracellular fragments, as well as Notch1/2 activation. The IL-4-induced increase in Notch1/2 activation was independent of the concomitant upregulated Jagged1 levels. Indeed, blocking Notch-Jagged1 interactions among CLL cells with Jagged1 neutralizing antibodies did not affect the expression of the Notch target Hes1. Notably, anti-Jagged1 antibodies partially prevented the IL-4-induced increase in Jagged1 processing and cell viability, suggesting that Jagged1 processing is one of the events contributing to IL-4-induced CLL cell survival. Consistent with this, Jagged1 silencing by small interfering RNA partially counteracted the capacity of IL-4 to promote CLL cell survival. Investigating the pathways whereby IL-4 promoted Notch1/2 activation in CLL cells independent of Jagged1, we found that PI3Kδ/AKT and PKCδ were involved in upregulating Notch1 and Notch2 proteins, respectively. Overall, this study provides new insights into the Notch-ligand system in CLL cells and suggests that targeting this system may be exploited as a novel/additional therapy approach for CLL.

## Introduction

The Notch receptor-ligand system mediates cell–cell communications and coordinates cell fate decisions in many contexts^1,2^. Notch signaling initiates in the signal-receiving cells when Notch receptors (Notch1–4) bind their ligands, from Jagged (Jag) or Delta-like (Dll) families, expressed on the signal-sending cells. This *trans*-interaction elicits two proteolytic cleavages of Notch receptors, resulting in the release and nuclear translocation of Notch intracellular domain (Notch-IC) and increased expression of target genes^3^.

Interestingly, following Notch engagement, some Notch ligands not only activate Notch signaling in signal-receiving cells, but also undergo intramembrane proteolysis in signal-sending cells^4,5^. This process induces the release of an intracellular domain of the ligand which, like Notch-IC, acts as a signaling molecule, entering the nucleus and inducing transcriptional activation with consequent cellular responses^6–10^. In this context, there is evidence that Jag1 intracellular domain (Jag1-IC) increases AP-1 transcription factor activity^6^, that drives the expression of several cancer-related genes. Jag1-IC also controls mRNA expression of Jag1 itself and Notch3, playing a key role in cellular transformation^10^ and neoplastic cell proliferation^11^, and representing a link between aberrant Jag1 expression and tumorigenesis. Consistent with these studies, Jag1 overexpression was associated with poor prognosis in several tumors^12–14^, including hematological malignancies^11^. Moreover, when Notch-ligand interactions occur within the same cell (*cis*-interactions)^15^, Jag1-IC can regulate Notch signaling, either negatively by favoring Notch-IC degradation^16^, or positively by increasing Notch-IC transcriptional activity^11^. This mechanism of Notch regulation is particularly important in those cells which co-express Notch receptors and Notch ligands and simultaneously receive and send Notch signals^17^.

Alterations of Notch receptor-ligand system are involved in the pathogenesis of several malignancies^18^, including chronic lymphocytic leukemia (CLL), a hematological disease characterized by accumulation of neoplastic B cells resistant to apoptosis^19^ due to genetic lesions^20^ and microenvironmental stimuli^21,22^.

A constitutive Notch1/2 activation sustaining cell survival through downstream positive effects on important antiapoptotic proteins, is a common event in CLL^23–25^. In up to 20% of CLL patients, Notch activation is further increased by *NOTCH1* PEST domain mutation^26,27^, a lesion associated with disease progression and chemorefractoriness^28–32^. Notably, Notch activation also occurs in CLL cells without *NOTCH1* mutation^23–25,33,34^, but the underlying mechanisms are poorly understood. It has been shown that a role for Notch activation in CLL cells is played by Notch ligands expressed on surrounding normal cells, including nurse-like and bone marrow stromal cells^26,35^. We previously showed that even CLL cells constitutively express Jagged1 ligand^23^, but its role in CLL cell biology has never been explored. Whether and how microenvironmental components other than Notch ligands, such as cytokines released by non-tumor cells, can influence the Notch-ligand system in CLL cells also remain to be defined.

A cytokine playing an important role in CLL is IL-4. It is associated with CLL progression^36,37^, protects CLL cells from spontaneous and drug-induced apoptosis^38–40^, and increases BCR signaling, a key driver of CLL pathogenesis^41,42^. Additionally, IL-4 is provided by different T-cell subsets in a lymph node microenvironment where CLL cells show hyperactivated Notch1^34,43^.

Based on all these observations, we investigated whether Jagged1 expressed in CLL cells undergoes proteolytic processing and/or is able to induce Notch activation through autocrine/paracrine loops, focusing on the effect that IL-4 may exert on the Notch-Jagged1 system in these cells. Results reveal novel regulatory mechanisms of the Notch-ligand system that may open new therapeutic horizons for CLL.

## Results

### Jag1 is constitutively processed in CLL cells and generates a fragment which localizes to the nucleus

**Fig. 1:**
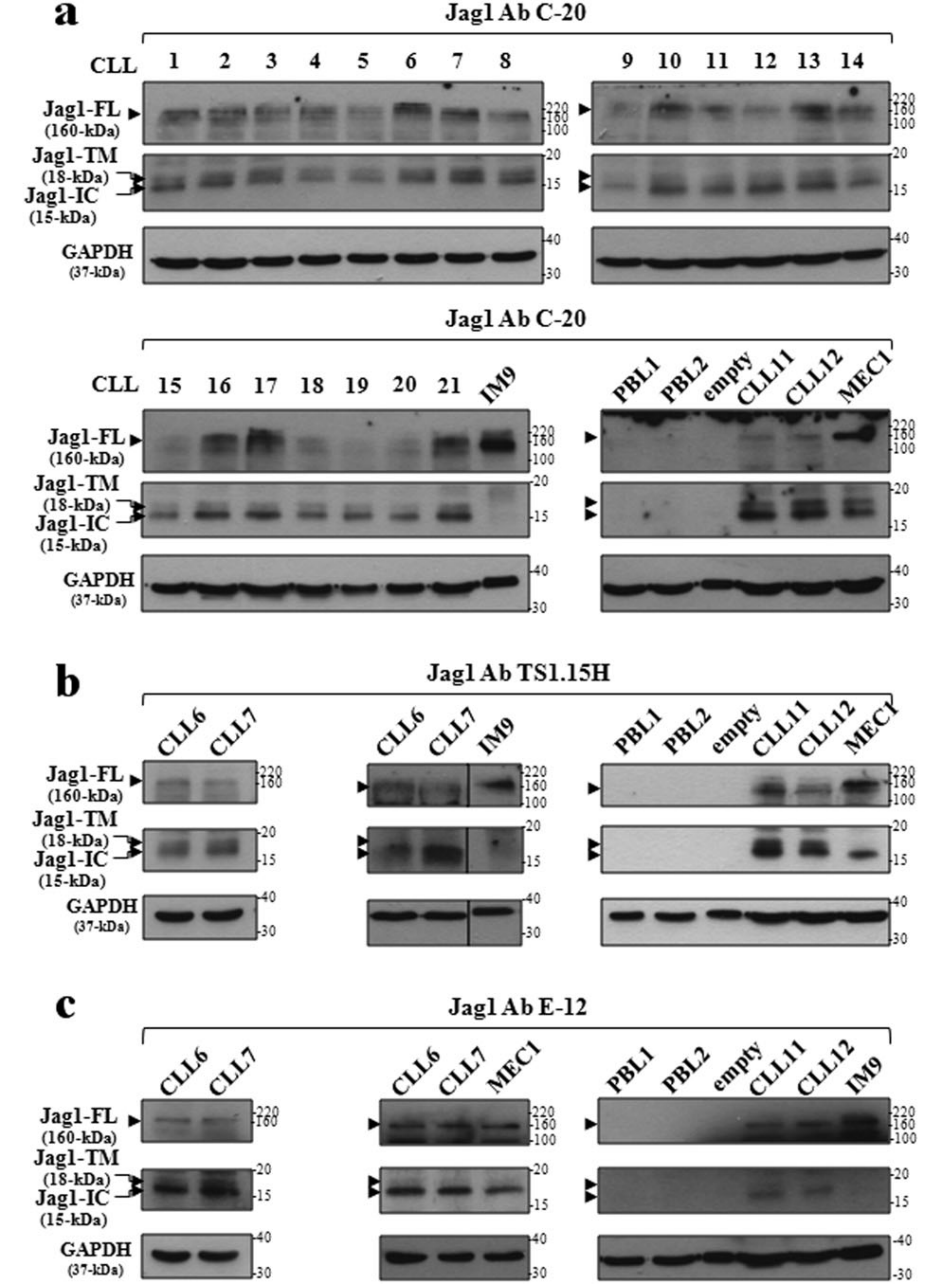
Jag1 is constitutively processed in CLL cells. **a**–**c** Western blot analysis of Jag1 was performed in 25 µg whole-cell lysates (WCL) from primary CLL cells (*n* = 21), MEC1 cell line, and PBL from healthy donors (*n* = 3), using three different C-terminal antibodies (Abs), as indicated on top of the blots. Data in panel **a** are obtained using the Jag1 C-20 Ab. Data in panels **b** and **c** are from representative CLL samples where the analysis of Jag1 was repeated using the Jag1 TS1.15H (**b**) and Jag1 E-12 (**c**) Abs. WCL isolated from IM9 cell line was used as a positive control for Jag1 expression. Protein loading was assessed reprobing the blots with an anti-GAPDH antibody. In each blot, separated panels are shown because different X-ray film exposures were necessary to detect Jag1-FL and Jag1 fragments. Positions of the molecular weight markers (kDa) are indicated on the right of the blots. In the blots with PBL, an empty lane has been placed between PBL2 and CLL11 to avoid cross-contamination. Vertical line inserted in the middle blot of panel **b** indicates a repositioned gel lane. Full images of all blots are shown in Supplementary Figure S1

In contexts other than CLL, Jag1 is processed by an ADAM-like activity liberating a soluble extracellular fragment (sJag1-EC) which regulates Notch signaling in neighboring cells^11,44^, and a membrane-associated fragment (Jag1-TM) which is cleaved by γ-secretase generating a transcriptionally active Jag1 domain (Jag1-IC)^6,10^. Thus, we analyzed Jag1 protein by Western blot (WB) in whole-cell lysates of primary CLL cells (*n* = 21), using three different antibodies, all directed against the Jag1 C-terminal. As shown in Fig. 1a and Supplementary Figure S1A, obtained using the indicated antibody, all samples showed a band of about 160-kDa corresponding to full-length Jag1 (Jag1-FL) as previously reported^23^, and two immunoreactive species at approximately 18 and 15 kDa. Consistent with previous studies on Jag1 cleaved fragments, these species correspond to the molecular weights predicted for Jag1-TM and Jag1-IC, respectively^6,44–46^. Indeed, sequence analysis of human Jag1 showed that putative cleavage site by ADAM activity resides within a hydrophobic stretch of amino acids (1048–1055) belonging to Jag1-juxtamembrane region^45,46^, whereas the putative cleavage site by γ-secretase corresponds to Val 1086^6^. The expression of Jag1-FL, Jag1-TM, and Jag1-IC was also detected in MEC1 cell line, established from peripheral blood leukemic B cells of a CLL patient, and widely used for studying this leukemia^47^ (Fig. 1a and Supplementary Figure S1A). Conversely, the expression of the three forms of Jag1 was absent in normal peripheral blood lymphocytes (PBL) from healthy donors (Fig. 1a and Supplementary Figure S1A), used as negative control of Jag1 expression^23,48^. All the above results were confirmed using two other anti-Jag1 C-terminal antibodies (Fig. 1b, c and Supplementary Figure S1B, C).

**Fig. 2:**
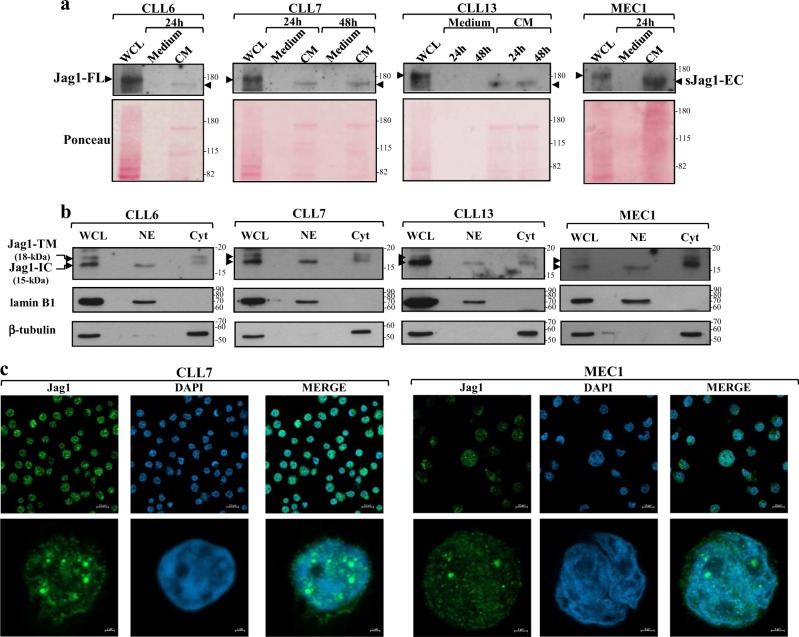
Jag1 processing generates an extracellular fragment released into CLL culture medium and an intracellular fragment that translocates into the nucleus. **a** Western blot analysis of sJag1-EC was performed, using the N-terminal Jag1 AF1277 antibody, in soluble proteins (40 µg) of conditioned medium (CM) collected from CLL cells (*n* = 10; CLL4-13, selected to include patients with different clinical and biological characteristics), and from MEC1 cell line cultured for the indicated times in serum-free conditions. Serum-free medium (indicated as medium) incubated in the same conditions of CLL and MEC1 cultures, but without leukemic cells, was used as negative control. WCL from each respective CLL sample and from MEC1 cell line were used as a control for the molecular weight of Jag1-FL. Ponceau red staining is shown to assess protein loading. Representative CLL cases are shown. Full images of the blots are shown in Supplementary Figure S2A. **b** Western blot analysis of Jag1 was performed, using the C-terminal Jag1 C-20 antibody, in 25 μg nuclear (NE) and cytoplasmic (Cyt) extracts from CLL (*n* = 10; CLL4-13) and MEC1 cells. Adequate fractionation and protein loading were assessed using anti-lamin B1 and anti-β-tubulin antibodies. WCL from each respective CLL sample and from MEC1 cells were used as a control for the molecular weight of Jag1-TM and Jag1-IC. An empty lane was placed between WCL, NE and Cyt lanes to avoid potential cross-contamination. Representative CLL cases are shown. Full images of the blots are shown in Supplementary Figure S2B. **c** Confocal microscopy images of subcellular localization of Jag1-IC in a representative CLL sample and in MEC1 cells. CLL cells (*n* = 5; CLL7-11) and MEC1 cells were stained with the C-terminal Jag1 HPA021555 antibody (green) and with DAPI for nuclei (blue), as described in “Materials and methods”, and then analyzed by confocal microscopy. Upper images show a representative field, lower images show one single slice from the z-stacks of a representative CLL or MEC1 cell in the field. Scale bars are indicated in each image

Consistent with the detection of Jag1-TM in CLL cells, we found that a sJag1-EC fragment was released from CLL cells into the cell culture supernatant referred to as conditioned medium (CM). WB analysis of soluble proteins in CM recovered from CLL cells cultured for 24 or 48 h in serum-free conditions showed a band compatible with the ADAM-cleaved sJag1-EC^44,45^, that migrated slightly faster than Jag1-FL detected in whole-cell lysates of CLL cells, and was absent in serum-free medium incubated without CLL cells, used as negative control (Fig. 2a and Supplementary Figure S2A). A band compatible with sJag1-EC was also detected in CM recovered from 24 h culture of MEC1 cells (Fig. 2a and Supplementary Figure S2A). To further investigate Jag1 processing in CLL cells, we examined subcellular localization of Jag1 fragments. WB analysis of nuclear and cytoplasmic extracts showed that whereas traces of Jag1-TM were found only in the cytoplasmic fraction, Jag1-IC was mainly detected in the nuclear extracts and to a lesser extent in the cytoplasm of CLL cells (Fig. 2b and Supplementary Figure S2B). Jag1-IC, but not Jag1-TM, was also present in nuclear extracts of MEC1 cells (Fig. 2b and Supplementary Figure S2B).

To better define the subcellular localization of Jag1-IC in CLL cells, we performed confocal immunofluorescence analysis. Consistent with WB data, immunofluorescence assay showed that Jag1-IC was mainly accumulated in the nucleus of CLL cells (Fig. 2c). Nuclear localization of Jag1-IC was also detected in MEC1 cells (Fig. 2c), whereas no expression of Jag1-IC was found in PBL (Supplementary Figure S3). Altogether, these results indicate constitutive Jag1 processing and nuclear translocation of Jag1-IC in CLL cells, suggesting a possible Jag1 intracellular signaling.

### Analysis of the correlation between Jag1 expression and clinical status of CLL patients

**Fig. 3:**
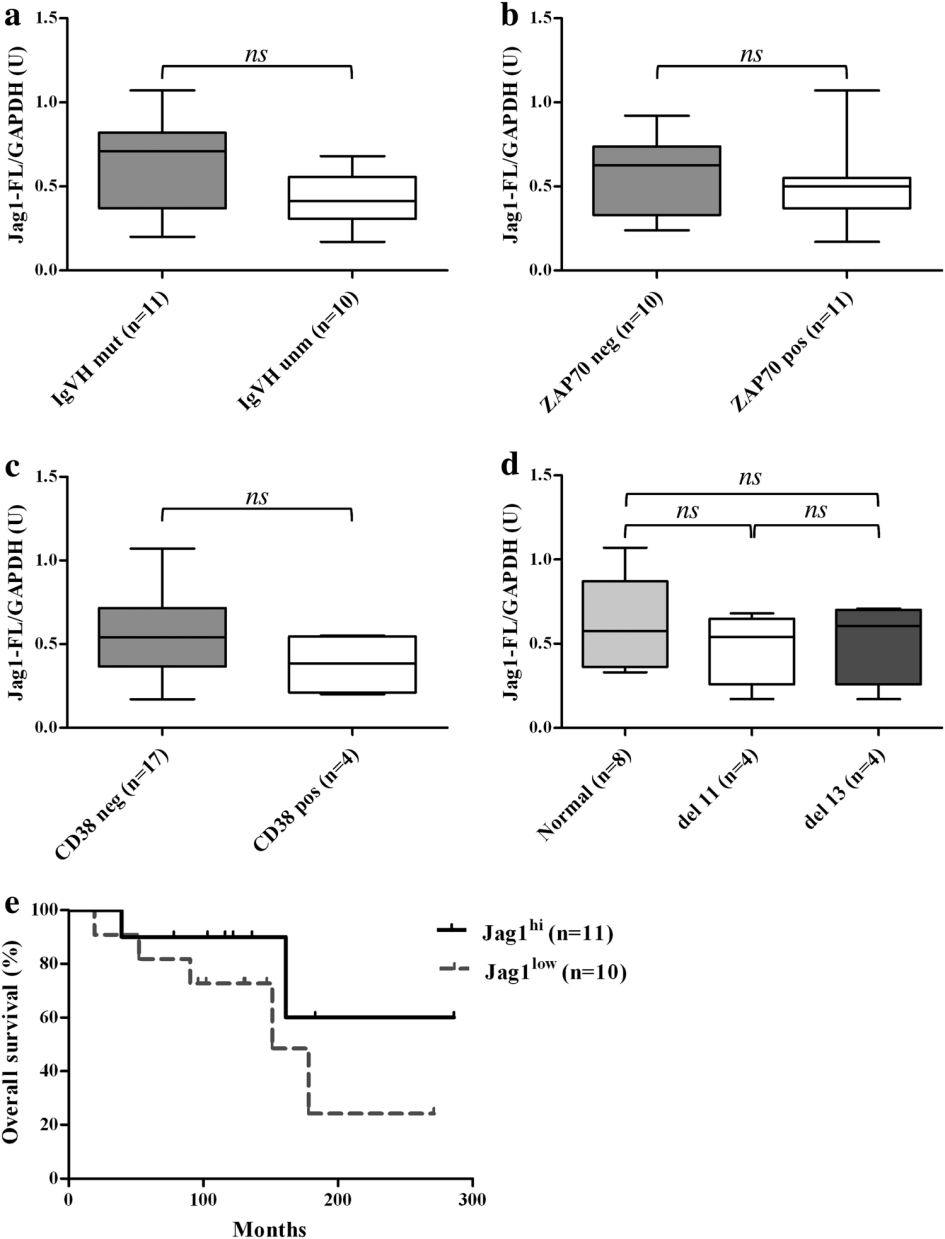
Analysis of the correlation between Jag1 expression levels and clinical status of CLL patients. **a**–**d** Comparison of Jag1-FL expression levels in CLL subgroup patients according to prognostic factors. The expression levels of Jag1-FL, calculated as a Jag1-FL/GAPDH ratio and expressed as densitometric units (U), were evaluated in the context of *IGVH* mutational status (**a**), ZAP70 expression (**b**), CD38 expression (**c**), and cytogenetic subgroups (**d**). Statistical analysis was performed using the Mann–Whitney test; *ns*: not significant. **e** Kaplan–Meier curves for overall survival (OS) in CLL patients according to Jag1-FL expression levels. CLL patients were divided into two subgroups: one subgroup (*n* = 11) defined as Jag1^hi^ with a Jag1-FL/GAPDH ratio ≥ 0.53; the other subgroup (*n* = 10) defined as Jag1^low^ with a Jag1-FL/GAPDH ratio < 0.53. Survival curves were compared using log-rank test. Differences were not significant

In order to shed light on the clinical significance of Jag1 expression in CLL, we examined whether there was a correlation between the expression levels of Jag1-FL in CLL cells and some CLL prognostic factors, including *IGVH* mutational status, expression of ZAP70 and CD38, and 11q and 13q deletions. Supplementary Table S1 gives the clinical and biological characteristics of CLL patients. Supplementary Table S2 shows, for each CLL sample, the expression levels of Jag1-FL normalized to GAPDH levels. Results in Fig. 3a–d showed that there were no significant differences in the Jag1-FL levels based on the analysis of the prognostic factors examined. We then investigated whether there was a correlation between Jag1-FL levels and the overall survival (OS) in CLL patients. Jag1-FL levels in all CLL samples ranged from 0.17 to 1.07 (Supplementary Table S2), and the mean and median values of the Jag1-FL/GAPDH ratio were both 0.53. We used this value as an arbitrary cut-off to divide CLL samples into Jag1^hi^ (Jag1-FL/GAPDH ratio ≥ 0.53) and Jag1^low^ (<0.53) subgroups. Results showed that there was no significant difference in OS rate between Jag1^hi^ and Jag1^low^ patients (Fig. 3e). Altogether, these analyses show that Jag1-FL expression is not correlated with the clinical status of CLL patients, but this might be due to the small sample size.

### IL-4 enhances Jag1 expression in CLL cells

**Fig. 4:**
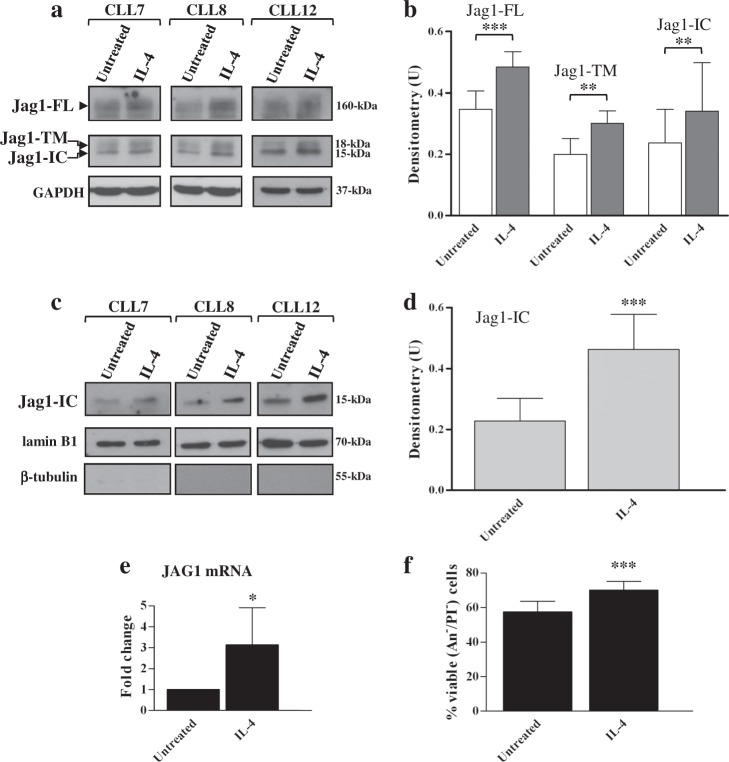
IL-4 enhances Jag1 expression in CLL cells. **a**–**f** CLL cells were cultured for 24 h with or without 25 ng/ml IL-4 (*n* = 6; CLL7-12, selected to include patients with different clinical and biological characteristics). **a**, **b** Western blot analysis of Jag1 was performed, using the C-terminal Jag1 C-20 antibody, in 25 µg whole-cell lysates, and protein loading was assessed reprobing the blots with an anti-GAPDH antibody. **a** In each blot, separated panels are shown because different X-ray film exposures were necessary to detect Jag1-FL and Jag1 fragments. Representative cases are shown. Full images of the blots are shown in Supplementary Figure S4A. **b** The density of the bands corresponding to Jag1-FL, Jag1-TM, and Jag1-IC was evaluated by densitometric analysis, and densitometry units (U) were calculated relative to GAPDH. Data are the mean ± SD of six patients. ^**^P < 0.01, ^***^P < 0.001 according to Student’s *t* test. **c**, **d** Western blot of Jag1-IC was performed, using the C-terminal Jag1 C-20 antibody, in 25 µg nuclear extracts. Protein loading and adequate fractionation were assessed using anti-lamin B1 and anti-β-tubulin antibodies. **c** Representative cases are shown. Full images of the cropped blots are shown in Supplementary Figure S4B. **d** Blots of each sample were subjected to densitometric analysis, and densitometry units (U) were calculated relative to lamin B1. Data are the mean ± SD of six patients. ^***^P < 0.001 according to Student’s *t* test. **e** mRNA levels of Jag1 were evaluated by quantitative real-time PCR, normalized to GAPDH and represented as fold change with respect to untreated cells. Results are the mean ± SD of six patients. ^*^P < 0.05 according to Student’s *t* test. **f** Cell viability and apoptosis were evaluated by flow cytometric analysis of Annexin V/PI (An V/PI) staining. Results are presented as percentage of viable (An V^−^/PI^−^) cells and are the mean ± SD of six patients. ^***^P < 0.001 according to Student’s *t* test

It is known that IL-4 sustains survival and activation of CLL cells, confers drug resistance and is associated with CLL progression^36–42^. Here, we analyzed the effect of IL-4 on Jag1 protein expression in CLL cells after 24 h treatment. Results showed that IL-4 enhanced expression of Jag1-FL, Jag1-TM and Jag1-IC, compared with untreated cells (Fig. 4a, b and Supplementary Figure S4A). Increased levels of Jag1-IC were also observed in nuclear extracts of IL-4-stimulated cells (Fig. 4c, d and Supplementary Figure S4B).

The effect of IL-4 on Jag1 occurred at transcriptional level, because quantitative real-time polymerase chain reaction (PCR) analysis showed that IL-4 increased Jag1 mRNA expression (Fig. 4e). Consistent with previous studies^38^, IL-4 increased CLL cell viability with effects evident at 24 h (Fig. 4f). Altogether, these results show that Jag1 expression is enhanced by a microenvironmental stimulus which simultaneously increases CLL cell survival.

### Effects of IL-4 on Jag1 expression in CLL cells are mediated by the PI3Kδ/AKT signaling

We next examined the signaling pathway involved in IL-4-induced Jag1 upregulation in CLL cells. It has been shown that IL-4 activates several signaling molecules in these cells, including JAK3/STAT6 and PI3K/AKT^38,41^. We investigated the PI3K/AKT pathway because it is strictly associated with CLL pathogenesis^49^, and PI3Kδ, one of the main activators of AKT in CLL cells^50^, is a therapeutic target in this leukemia^51,52^.

**Fig. 5:**
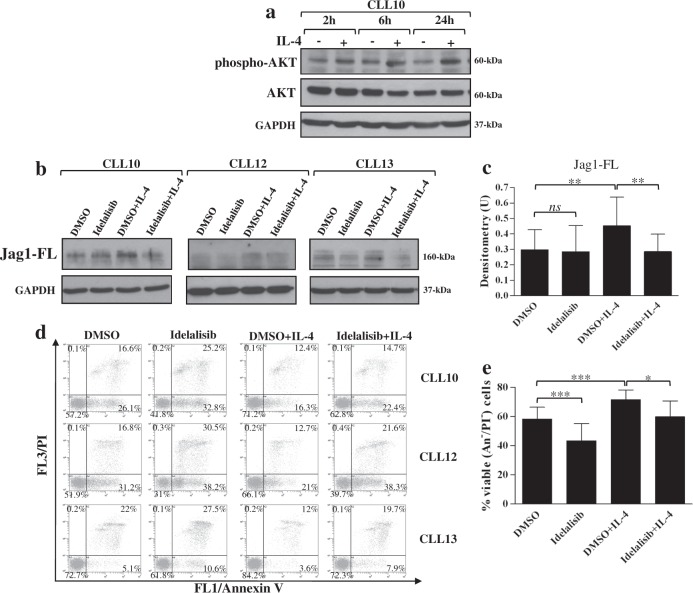
Effects of IL-4 on Jag1 expression in CLL cells are mediated by the PI3Kδ/AKT signaling. **a** Western blot analysis of AKT Ser473 phosphorylation (phospho-AKT) was performed in 25 µg whole-cell lysates from CLL cells cultured with or without 25 ng/ml IL-4 for the indicated times. Protein loading was assessed using anti-total AKT and anti-GAPDH antibodies. One representative case is shown. Full image of the blot is shown in Supplementary Figure S5A. **b**–**e** CLL cells, pretreated for 2 h with the PI3Kδ inhibitor Idelalisib (5 µM) or 0.01% DMSO as control, were incubated for further 24 h with or without 25 ng/ml IL-4 (*n* = 6; CLL10 and CLL12-16, selected to include patients with different clinical and biological characteristics). **b** Western blot analysis of Jag1 was performed, using the C-terminal Jag1 C-20 antibody, in 25 µg whole-cell lysates, and protein loading was assessed reprobing the blots with an anti-GAPDH antibody. Representative cases are shown. Full images of the blots are shown in Supplementary Figure S5B. **c** Blots of each sample were subjected to densitometric analysis, and densitometry units (U) were calculated relative to GAPDH. Data are the mean ± SD of six patients. *ns*: the difference between the two groups was not significant; ^**^P < 0.01 according to Student’s *t* test. **d**, **e** Cell viability and apoptosis were evaluated by flow cytometric analysis of Annexin V/PI (An V/PI) staining. **d** Results are presented as the percentage of viable (An V^−^/PI^−^), early apoptotic (An V^+^/PI^−^), late apoptotic (An V^+^/PI^+^), and necrotic (An V^−^/PI^+^) cells. Representative cases are shown. **e** Results are presented as percentage of viable (An V^−^/PI^−^) cells and are the mean ± SD of six patients. ^*^P < 0.05, ^***^P < 0.001 according to Student’s *t* test

We first examined the effect of IL-4 on AKT Ser473 phosphorylation as a readout for PI3Kδ activation, and found that the basal AKT phosphorylation began to increase at 2 h and continued until 24 h (Fig. 5a and Supplementary Figure S5A). Next, CLL cells were pretreated for 2 h with the PI3Kδ inhibitor Idelalisib or DMSO as control, and then incubated for additional 24 h with IL-4 before analysis of Jag1 protein. Results showed that Idelalisib completely prevented IL-4-induced increase in Jag1 expression and had a very marginal effect on constitutive Jag1 levels (Fig. 5b, c and Supplementary Figure S5B). As expected^51^, Idelalisib reduced viability of control CLL cells and prevented IL-4-induced increase in cell viability (Fig. 5d, e).

### IL-4 enhances Notch1 and Notch2 expression and activation in CLL cells

We previously reported that IL-4, after 72 h treatment, increased expression/activation of Notch1/2 receptors while promoting CLL cell survival^25^. Here, we analyzed the effect of IL-4 treatment for 24 h on Notch1 and Notch2 proteins in the same group of CLL samples where IL-4 had increased Jag1 expression.

**Fig. 6:**
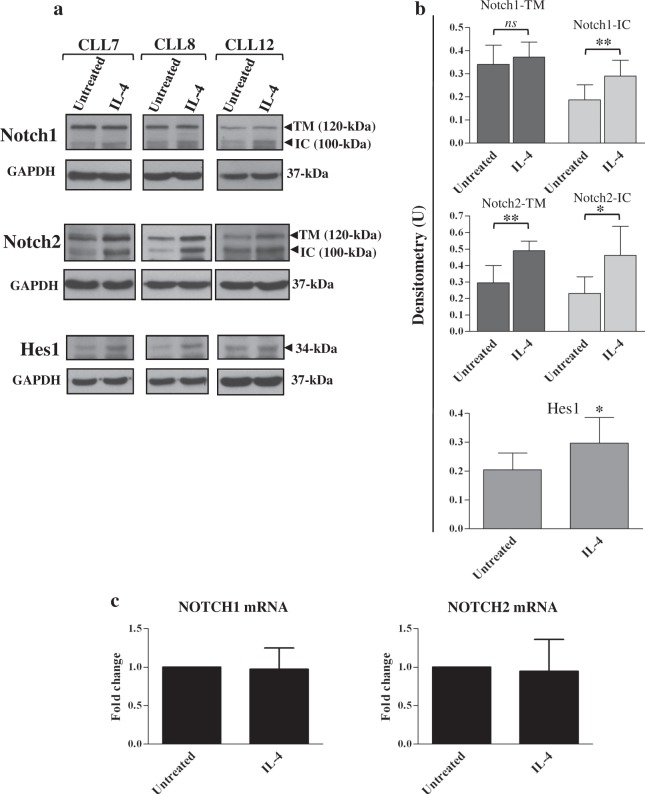
IL-4 enhances Notch1 and Notch2 expression and activation in CLL cells. **a**–**c** CLL cells were cultured for 24 h with or without 25 ng/ml IL-4 (*n* = 6; CLL7-12, selected to include patients with different clinical and biological characteristics). **a**, **b** Western blot analysis of Notch1, Notch2, and Hes1 was performed in 25 µg whole-cell lysates, and protein loading was assessed using an anti-GAPDH antibody. **a** Representative cases are shown. Full images of the blots are shown in Supplementary Figure S6A–C. **b** The density of the bands corresponding to Notch1-TM, Notch1-IC, Notch2-TM, Notch2-IC, and Hes1 was evaluated by densitometric analysis, and densitometry units (U) were calculated relative to GAPDH. Data are the mean ± SD of six patients. *ns*: the difference between the two groups was not significant; ^*^P < 0.05, ^**^P < 0.01 according to Student’s *t* test. **c** Notch1 and Notch2 mRNA levels were evaluated by real-time PCR, normalized to GAPDH and represented as fold change with respect to untreated cells. Results are the mean ± SD of six patients. The effects of IL-4 are not significant

Analysis of Notch1 showed that IL-4 mainly increased the levels of Notch1-IC, and had a marginal effect on Notch1-TM (Fig. 6a, b and Supplementary Figure S6A). Analysis of Notch2 showed that IL-4 enhanced the expression of both Notch2-TM and Notch2-IC at similar extent (Fig. 6a, b and Supplementary Figure S6B). The effect of IL-4 on Notch1 and Notch2 proteins was accompanied by enhanced Notch activation, as revealed by the increased protein expression of the Notch target Hes1 (Fig. 6a, b and Supplementary Figure S6C). Conversely, quantitative real-time PCR analysis showed that mRNA levels of both Notch receptors were not affected by IL-4 (Fig. 6c), suggesting that IL-4 regulates Notch receptors at the posttranscriptional level. Therefore, in CLL cells, Jag1 expression and Notch1/2 expression/activation were concomitantly upregulated by IL-4.

### Blocking Notch-Jag1 interactions among CLL cells does not affect IL-4-induced Notch activation but reduces IL-4-induced Jag1 processing and CLL cell viability

**Fig. 7:**
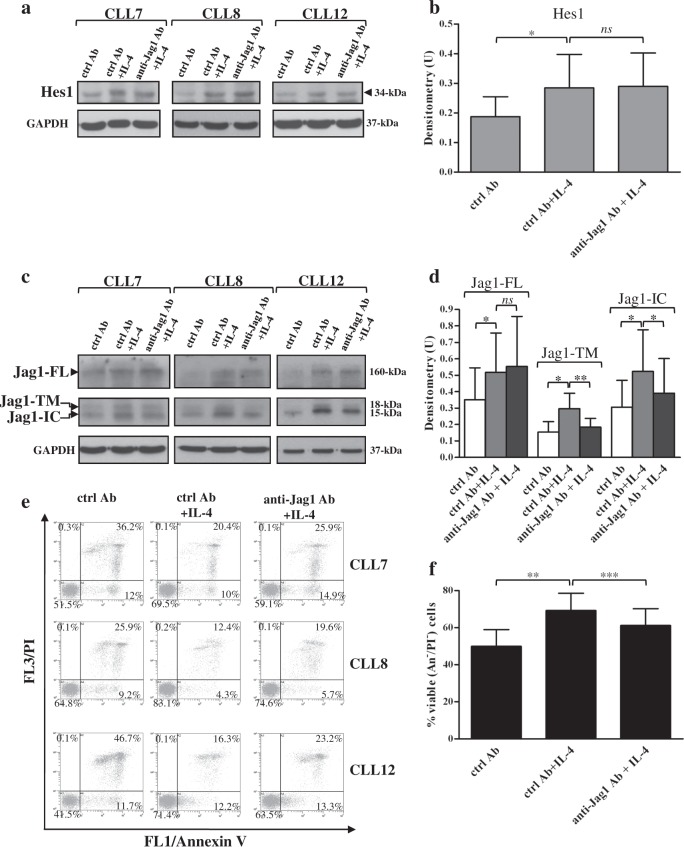
Blocking Notch–Jag1 interactions among CLL cells does not affect IL-4-induced Notch activation, but reduces IL-4-induced Jag1 processing and cell viability. **a**–**f** CLL cells were cultured for 48 h with or without 25 ng/ml IL-4 in the presence of 60 µg/ml Jag1 neutralizing or goat IgG antibodies as isotype control (ctrl Ab) (*n* = 5; CLL7-8 and CLL10-12, selected to include patients with different clinical and biological characteristics). **a**–**d** Western blot analysis of Hes1 and Jag1 was performed in 25 µg whole-cell lysates, and protein loading was assessed reprobing the blots with an anti-GAPDH antibody. **a**, **c** Representative cases are shown. **a** Full images of Hes1 blots are shown in Supplementary Figure S7A. **c** For Jag1 analysis, the C-20 antibody was used. In each blot, separated panels are shown because different X-ray film exposures were necessary to detect Jag1-FL and Jag1 fragments. Full images of Jag1 blots are shown in Supplementary Figure S7B. **b**, **d** The density of the bands corresponding to Hes1, Jag1-FL, Jag1-TM, and Jag1-IC was evaluated by densitometric analysis, and densitometry units (U) were calculated relative to GAPDH. Data are the mean ± SD of five patients. *ns*: the difference between the two groups was not significant; ^*^P < 0.05, ^**^P < 0.01 according to Student’s *t* test. **e**, **f** Cell viability and apoptosis were evaluated by flow cytometric analysis of Annexin V/PI (An V/PI) staining. **e** Results are presented as the percentage of viable (An V^−^/PI^−^), early apoptotic (An V^+^/PI^−^), late apoptotic (An V^+^/PI^+^), and necrotic (An V^−^/PI^+^) cells. Representative cases are shown. **f** Results are presented as percentage of viable (An V^−^/PI^−^) cells and are the mean ± SD of five patients. ^**^P < 0.01, ^***^P < 0.001 according to Student’s *t* test

Based on the above evidence, we analyzed whether in IL-4-stimulated CLL cells, the increased Jag1 levels could be responsible for the increased Notch activation, favoring Notch–Jag1 interactions among CLL cells. We blocked these interactions culturing cells for 48 h with IL-4 in the presence of Jag1 neutralizing or isotype control antibodies, and then we analyzed Hes1 protein expression. Results showed that anti-Jag1 antibodies did not prevent the IL-4-induced increase in Hes1 levels, suggesting that IL-4-induced Notch activation does not depend on the increased availability of Jag1 in CLL cells (Fig. 7a, b and Supplementary Figure S7A).

As Notch-ligand interactions induce activation not only of Notch receptors but even of Jag1 ligand^6,9,11^, we examined whether in CLL cells cultured with IL-4, blocking Notch–Jag1 interactions could affect Jag1 processing. Results showed that anti-Jag1 antibodies partially prevented IL-4-induced increase in Jag1-TM and Jag1-IC levels without affecting the increase of Jag1-FL (Fig. 7c, d and Supplementary Figure S7B). Next, because in some tumors, Jag1 signaling sustains tumor growth^11^, we analyzed whether upon IL-4 treatment, the decreased levels of Jag1 fragments, observed after blocking Notch–Jag1 interactions, could be accompanied by reduced cell viability. Results showed that anti-Jag1 antibodies partially prevented the IL-4-induced increase in cell viability (Fig. 7e, f). Altogether, these results suggest that Notch-Jag1 interactions among CLL cells favor Jag1 processing, and that Jag1 processing and CLL cell survival are events associated each other.

### Jag1 silencing counteracts the IL4-dependent increase of CLL cell viability

**Fig. 8:**
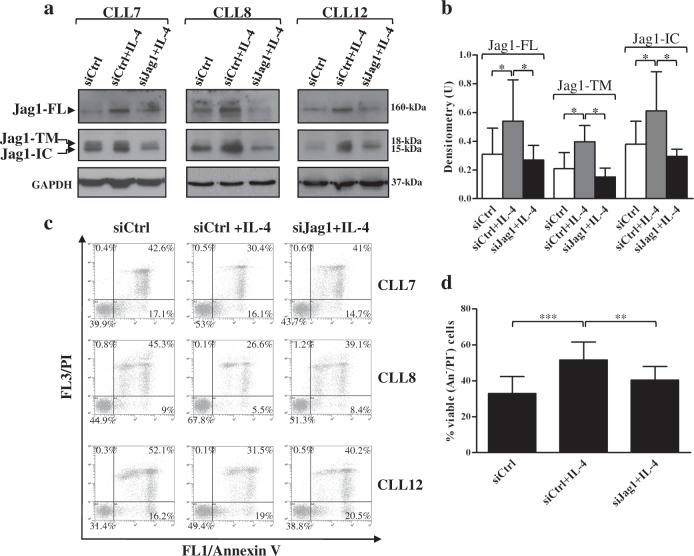
Jag1 silencing counteracts the IL4-dependent increase of CLL cell viability. **a**–**d** CLL cells were transfected with control siRNA (siCtrl) and Jag1 siRNA (siJag1), as described in “Materials and methods”, and then were cultured for 72 h in complete medium containing 25 ng/ml IL-4 (*n* = 5; CLL7-8 and CLL10-12). **a** Western blot analysis of Jag1 was performed, using the Jag1 C-20 antibody, in 25 µg whole-cell lysates, and protein loading was assessed reprobing the blots with an anti-GAPDH antibody. Representative cases are shown. In each blot, separated panels are shown because different X-ray film exposures were necessary to detect Jag1-FL and Jag1 fragments. Full images of the blots are shown in Supplementary Figure S8. **b** The density of the bands corresponding to Jag1-FL, Jag1-TM, and Jag1-IC was evaluated by densitometric analysis, and densitometry units (U) were calculated relative to GAPDH. Data are the mean ± SD of five patients. ^*^P < 0.05 according to Student’s *t* test. **c**, **d** Cell viability and apoptosis were evaluated by flow cytometric analysis of Annexin V/PI (An V/PI) staining. **c** Results are presented as the percentage of viable (An V^−^/PI^−^), early apoptotic (An V^+^/PI^−^), late apoptotic (An V^+^/PI^+^), and necrotic (An V^−^/PI^+^) cells. Representative cases are shown. **d** Results are presented as percentage of viable (An V^−^/PI^−^) cells and are the mean ± SD of five patients. ^**^P < 0.01, ^***^P < 0.001 according to Student’s *t* test

To better investigate the functional role of Jag1 in the IL-4-dependent increase of CLL cell viability, we examined whether downregulating Jag1 protein using small interfering RNA (siRNA) could affect this event. CLL cells were transfected with control nontargeting (siCtrl) or Jag1 siRNA (siJag1) and then cultured for 72 h with IL-4. WB analysis of Jag1 showed that upon IL-4 treatment, the increased expression of Jag1-FL and Jag1 fragments observed in siCtrl cells, was reduced in siJag1 cells, indicating siJag1 transfection efficiency (Fig. 8a, b and Supplementary Figure S8). Downregulation of Jag1 expression/activation was accompanied by a partial reversal of the IL-4-induced increase of viability observed in siCtrl cells (Fig. 8c, d), strengthening the hypothesis that Jag1 participates in sustaining the IL-4-dependent CLL cell survival.

### Signaling pathways involved in Notch1 and Notch2 upregulation induced by IL-4 in CLL cells

**Fig. 9:**
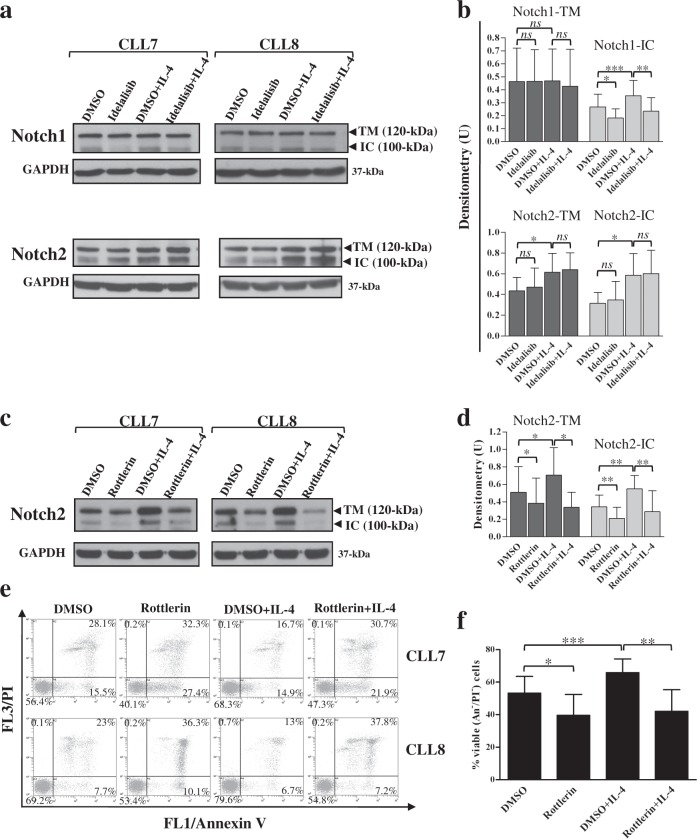
Signaling pathways involved in Notch1 and Notch2 upregulation induced by IL-4 in CLL cells. CLL cells (*n* = 5; CLL7-8 and CLL10-12, selected to include patients with different clinical and biological characteristics) were preincubated for 2 h with 5 µM Idelalisib or 0.01% DMSO (**a**, **b**), or with 10 µM Rottlerin or 0.05% DMSO (**c**–**f**), and then were cultured with or without 25 ng/ml IL-4 for further 24 h. **a–d** Western blot analysis of Notch1 and Notch2 was performed in 25 µg whole-cell lysates, and protein loading was assessed using an anti-GAPDH antibody. **a**, **c** Representative cases are shown. Full images of the blots are shown in Supplementary Figure S9A–C. **b**, **d** The density of the bands corresponding to Notch1-TM, Notch1-IC, Notch2-TM, and Notch2-IC was evaluated by densitometric analysis, and densitometry units (U) were calculated relative to GAPDH. Data are the mean ± SD of five patients. *ns*: the difference between the two groups was not significant; ^*^P < 0.05, ^**^P < 0.01, ^***^P < 0.001 according to Student’s *t* test. **e**, **f** Cell viability and apoptosis were evaluated by flow cytometric analysis of Annexin V/PI (An V/PI) staining. **e** Results are presented as the percentage of viable (An V^−^/PI^−^), early apoptotic (An V^+^/PI^−^), late apoptotic (An V^+^/PI^+^), and necrotic (An V^−^/PI^+^) cells. Representative cases are shown. **f** Results are presented as percentage of viable (An V^−^/PI^−^) cells and are the mean ± SD of five patients. ^*^P < 0.05, ^**^P < 0.01, ^***^P < 0.001 according to Student’s *t* test

The above evidence that the IL-4-induced increase in Notch1/2 activation was independent of Jag1 upregulation prompted us to investigate the molecular pathways responsible for this event. It has been shown that in T-ALL cells, a role in promoting deregulated Notch1 activation is played by an excessive PI3K/AKT signaling^53^. Based on these observations and given the hyperactivation of PI3Kδ/AKT signaling in CLL^38,49^ and our evidence that IL-4 further increased PI3Kδ/AKT activity, we examined whether inhibiting this pathway could contrast the stimulatory effect of IL-4 on Notch1 expression. CLL cells were preincubated with Idelalisib or DMSO for 2 h, and then cultured with IL-4 for additional 24 h, before WB analysis of Notch1. Idelalisib completely prevented IL-4-induced increase in Notch1-IC levels, and even reduced the constitutive Notch1-IC levels. Conversely, Idelalisib did not affect Notch1-TM expression in either unstimulated or IL-4-stimulated cells (Fig. 9a, b and Supplementary Figure S9A). When we analyzed the effect of Idelalisib on IL-4-induced increase in Notch2 expression, we found that Idelalisib affected neither constitutive nor IL-4-induced Notch2-TM and Notch2-IC levels (Fig. 9a, b and Supplementary Figure S9B). Altogether, these results suggest that in CLL cells, PI3Kδ/AKT contributes to sustaining both basal and IL-4-induced levels of Notch1-IC, whereas it is not involved in Notch2 regulation in either unstimulated and IL-4-stimulated CLL cells. It has been reported that in CLL cells, Notch2 is regulated by PKCδ^54^, a kinase constitutively active in these cells and important for their survival^55^. Furthermore, PKCδ has been shown to be involved in IL-4 signaling in normal human B cells^56^. On this basis, we investigated whether in CLL cells, the effect of IL-4 on Notch2 expression could be mediated by this kinase. We examined whether pretreatment of CLL cells with the PKCδ inhibitor Rottlerin for 2 h interfered with the increased expression of Notch2-TM and Notch2-IC induced by IL-4 at 24 h. Rottlerin completely prevented Notch2-TM and Notch2-IC upregulation induced by IL-4 and even reduced their basal levels (Fig. 9c, d and Supplementary Figure S9C), suggesting that PKCδ is involved in both constitutive and IL-4-induced Notch2 expression. Analysis of cell viability/apoptosis showed that Rottlerin reduced viability of control CLL cells as previously reported^55^, and continued to have proapoptotic effect even in the presence of IL-4 (Fig. 9e, f).

## Discussion

This study provides novel insights into the Notch-ligand system in CLL cells. We demonstrated that Jag1 is constitutively expressed and processed in these cells and generates a Jag1-IC fragment which translocates into the nucleus, suggesting the existence of a Jag1 intracellular signaling with a potential role in CLL cell biology. Expression/processing of Jag1 appears as a common event in CLL cells as it has been detected in all examined samples. However, no correlation was found between expression levels of Jag1 in CLL cells and OS in CLL patients. Jag1 expression levels are also unrelated with some risk-factors, including *IGVH* mutational status, ZAP70 and CD38 expression, and 11q and 13q deletions. Even the two samples carrying *NOTCH1* mutation (CLL8 and CLL13) show Jag1 levels comparable with those of *NOTCH1*-unmutated samples, suggesting that Jag1 expression is not influenced by *NOTCH1* mutation, at least when it is present in a small fraction of leukemic cells, as indicated by the low *NOTCH1* mutant allele burden detected in the two samples (Supplementary Table S1). The lack of correlation between Jag1 expression and the clinical status of CLL patients might be due to the small number of examined patients, and further analysis should be performed in larger patient cohorts.

We showed that detection of Jag1 intracellular fragments in CLL cells is accompanied by the presence, in the CM of CLL cultures, of a soluble extracellular Jag1 fragment, known to be released after the first cleavage of Jag1 by ADAM metalloproteinase^44,46^. These results are in keeping with other studies showing the presence of soluble Jag1 in the plasma from CLL patients^26^.

Nuclear translocation of Jag1-IC, an indicator of Jag1 signaling activation, has been previously found in different types of normal and cancer cells, where it controls gene expression modifying cell fate or phenotype^6–11,16^. However, whereas in some of these cell systems, Jag1 processing occurs after cell transfection with engineered Jag1 fragments^6,10,16^, in CLL cells, Jag1 processing occurs physiologically. Furthermore, IL-4, a microenvironmental factor important for CLL cell survival and pathogenesis^36–42^, increases Jag1 expression and the levels of nuclear Jag1-IC. Several signaling pathways have been shown to enhance Jag1 expression while promoting cancer^57^, including Notch3 in a mouse model of T-ALL^11^, epidermal growth factor receptor in non–smallcell lung cancer^58^, and Wnt/β-catenin in colorectal^59^ and ovarian cancer^60^. We show that in CLL cells, IL-4-induced upregulation of Jag1 protein is mediated by the PI3Kδ/AKT pathway, an important therapeutic target in this leukemia^49–52^.

As reported in our previous studies^25^ and confirmed here, IL-4 also enhances Notch1 and Notch2 expression in CLL cells. Specifically, IL-4 increases Notch1-IC levels and has a marginal effect on Notch1-TM, whereas both Notch2-TM and Notch2-IC are enhanced by this cytokine. IL-4-induced Notch1/2 expression is also accompanied by increased Notch activation, as indicated by higher levels of Hes1 protein. These results suggest that besides Notch ligands expressed on neighboring nontumor cells, as reported until now^26,35^, even other microenvironmental factors, such as cytokines, might potentiate Notch1/2 activation in CLL cells. In order to determine whether IL-4-induced Notch1/2 activation was due to the concomitant upregulated levels of Jag1 in CLL cells, we performed neutralization studies using anti-Jag1 antibodies, able to block Notch-Jag1 interactions among CLL cells. These antibodies fail to reduce IL-4-induced Hes1 levels, suggesting that IL-4 increases Notch signaling through Jag1-independent mechanisms. A possible explanation for the evidence that Jag1 does not contribute to IL-4-induced Notch signaling is that Notch-Jag1 interactions among CLL cells might occur either in *trans* or in *cis*, and therefore trigger either activation^3^ or inhibition of Notch signaling^15^. In such a case, these opposing signals might not lead to any evident Notch activation in CLL cells, so that, anti-Jag1 antibodies, by blocking both types of Notch-Jag1 interactions, might not have any evident effect. However, interestingly, anti-Jag1 antibodies partially prevented the IL-4-induced increase in Jag1 processing, suggesting that Notch–Jag1 interactions among CLL cells are important for this event, although they do not contribute to Notch activation. The impaired Jag1 processing induced by anti-Jag1 antibodies is also accompanied by reduction in IL-4-induced CLL cell viability, suggesting that Jag1 processing may be one of the events whereby IL-4 promotes CLL cell survival. Consistent with these data, Jag1 silencing by siRNA partially counteracts the capacity of IL-4 to promote CLL cell survival, strengthening the hypothesis that Jag1 participates in sustaining IL-4-dependent CLL cell survival, although further studies are necessary to define the underlying mechanisms.

In keeping with our hypothesis of a ligand-independent mechanism for IL-4-induced Notch1/2 activation in CLL cells, increased evidence has demonstrated in other cell types, that Notch is activated through alterations in Notch endosomal trafficking, recycling, ubiquitination, and degradation^61–65^. Several of these events regulating Notch activation are in turn regulated by signaling pathways often aberrantly activated in tumor cells. In T-ALL cells, hyperactive PI3K/AKT signaling deregulates Notch1 activation inhibiting its lysosome-mediated degradation^53^. In breast cancer stem cells, high levels of prolyl-isomerase Pin1 sustain Notch signaling protecting Notch1 and Notch4 from proteasomal degradation^66^. Here, we show that in CLL cells, PI3Kδ/AKT signaling contributes to sustaining IL-4-induced increase in Notch1-IC levels, whereas PKCδ signaling is involved in upregulating Notch2-TM and Notch2-IC levels, although the mechanisms whereby these pathways induce Notch deregulation remain undefined.

**Fig. 10:**
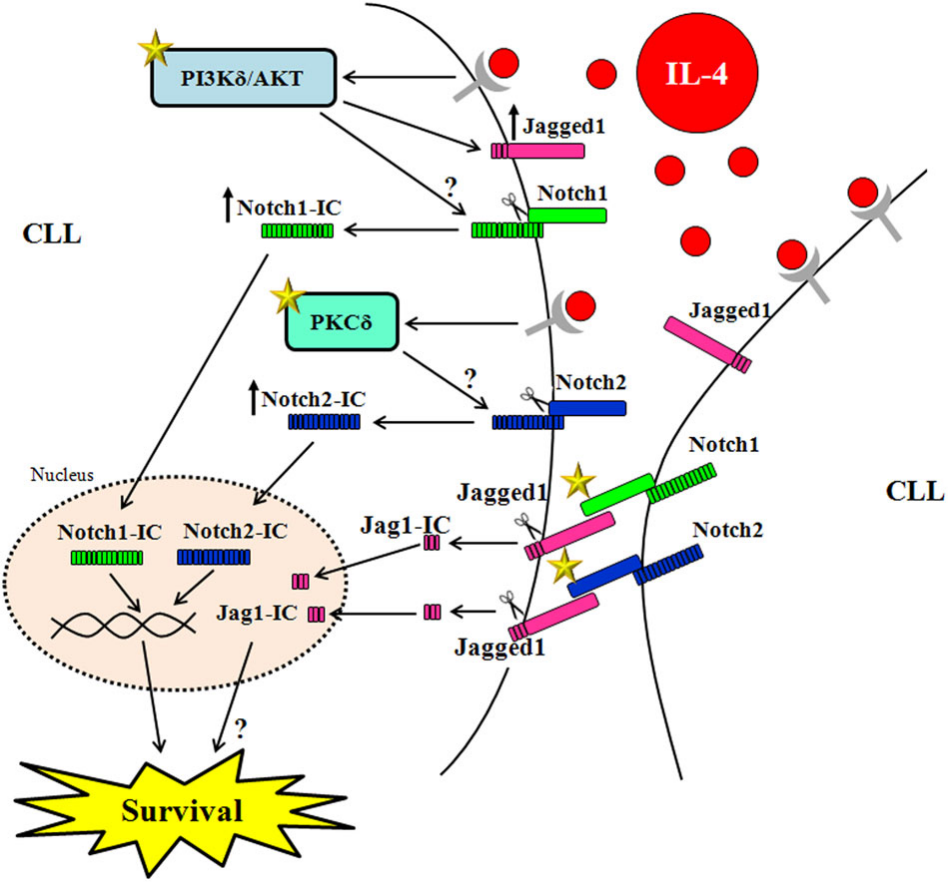
Schematic representation of the signaling network of Jag1 and Notch1/Notch2 in CLL cells in the presence of the prosurvival factor IL-4. CLL cells constitutively express both Notch1/Notch2 receptors and Jag1 ligand. IL-4 activates the PI3Kδ/AKT signaling to increase Jag1 expression and Notch1 activation, and the PKCδ signaling to increase Notch2 activation. Interactions between Jag1 and Notch1/Notch2 do not activate Notch signaling but induce Jag1 processing, generating an intracellular fragment (Jag1-IC) that migrates into the nucleus and contributes to CLL cell survival through mechanisms which remain to be defined (“?”). Even the mechanisms by which PI3Kδ/AKT and PKCδ activate Notch signaling in a ligand-independent manner are still not elucidated (“?”). The star symbols indicate potential druggable molecular events for targeting the Notch-ligand system in CLL

In conclusion, the evidence that in CLL cells, Jag1 is processed and associated with their survival, and Notch1/2 activation occurs independently of Jag1 expressed on these cells, suggests the rationale for new therapeutic approaches targeting the Notch-ligand system in CLL (Fig. 10). We suggest that a novel/additional therapeutic strategy may involve the combinatorial use of anti-Jag1 antibodies and inhibitors of PI3Kδ/AKT and PKCδ. The anti-Jag1 antibodies may prevent Jag1 signaling and possibly, Notch activation induced by Jag1 expressed on nontumor cells, and the kinase inhibitors may prevent Notch signaling activated independently of Jag1.

Given the growing evidence that Notch signaling is activated in a large fraction of CLL patients^23–25,33^, and given this study showing that even Jag1 processing is a common event in CLL, the identification of novel therapeutic strategies targeting the Notch-ligand system in CLL may have important implications for improving the management of this disease.

## Materials and methods

### Primary CLL samples and MEC1 CLL cell line

Twenty-one CLL patients entered this study. Diagnoses of CLL were based on Stanford criteria defined by the National Cancer Institute-sponsored Working Group^67^ and clinical staging was based on the Binet classification^68^. This study was approved by the local Ethics Committee, and all patients signed informed consent in accordance with the Declaration of Helsinki. CD19^+^/CD5^+^ CLL cells were isolated from peripheral blood samples as previously reported^23^. All CLL samples contained more than 95% CD19^+^/CD5^+^ CLL cells, as assessed by flow cytometry (EPICS-XL-MCL; Beckman Coulter, Fullerton, CA, USA). Normal PBL were isolated from healthy donors as previously reported.^23^ The human CLL MEC1 cell line was obtained from the German Collection of Microorganisms and Cell Cultures (DSMZ, Braunschweig, Germany), and cultured in Iscove’s MDM containing 10% fetal bovine serum.

### CLL clinical laboratory characteristics

*IgV*_*H*_ mutations, CD38 and ZAP70 expression, cytogenetic abnormalities, *NOTCH1* exon 34 mutation and the percentage of mutant allele burden were analyzed as reported^29,32^. Supplementary Table S1 gives the clinical and biological characteristics of CLL patients.

### Cell culture and treatments

CLL cells were cultured at 2 × 10^6^/ml in complete medium as previously described^23,25^. For stimulation studies, recombinant human IL-4 (Immunotools, Friesoyte, Germany) was used at 25 ng/ml. In signal transduction studies, CLL cells were pretreated for 2 h with the PI3Kδ inhibitor Idelalisib (CAL-101, 5 µM; Selleck Chemicals, Houston, TX, USA), the PKCδ inhibitor Rottlerin (10 µM; Calbiochem, La Jolla, CA, USA) or DMSO as control, before culture with IL-4 for further 24 h. For neutralization studies, CLL cells were cultured in 96-well plates at 2 × 10^5^/well in 200 µl complete medium containing 60 µg/ml human anti-Jag1 (AF1277) or normal goat IgG control (AB-108-C), all from R&D Systems (Minneapolis, MN, USA).

### Analysis of cell viability/apoptosis

Cell viability/apoptosis was assessed by flow cytometry (EPICS-XL-MCL) after Annexin V^-^FITC/PI staining, performed using a commercial kit (Immunotech, Beckman Coulter), according to manufacturer’s instructions.

### Whole-cell lysate extraction and subcellular protein fractionation

Whole-cell lysates were extracted as reported^23^. Nuclear- and cytoplasmic-enriched fractions were prepared using the NE-PER extraction kit (Thermo Fisher Scientific, Pierce Chemical, Rockford, IL, USA), according to manufacturer’s instructions.

### Analysis of Jag1 extracellular domain

The extracellular soluble Jag1 domain was analyzed in CM collected from CLL cells or MEC1 cell line cultured at 2 × 10^6^/ml in serum-free medium for 24 and 48 h. CM was centrifuged at 3000 x *g* for 10 min at 4 °C and concentrated using Vivaspin 6 with 50-kDa cut-off (Sartorius, Göttingen, Germany). After adding protease inhibitor cocktail 50X (Sigma-Aldrich, St. Louis, MO, USA), soluble proteins were subjected to Western blot analysis.

### Western blot

Western blot was performed as reported,^23^ by using the following primary antibodies: polyclonal anti-Jag1 C-terminal (C-20: sc-6011); monoclonal anti-Jag1 C-terminal (E-12: sc-390177); monoclonal anti-Hes1 (E-5: sc-166410), all from Santa Cruz Biotechnology (Santa Cruz, CA, USA); monoclonal anti-Jag1 C-terminal (clone TS1.15H), anti-Notch1 (clone bTAN20) and anti-Notch2 (clone C651.6DbHN), all developed by Spyros Artavanis-Tsakonas, obtained from DSHB and maintained at Iowa University; polyclonal anti-Jag1 N-terminal (AF1277) from R&D Systems; polyclonal anti-lamin B1 (ab16048) from Abcam (Cambridge, MA, USA); polyclonal anti-total AKT and anti-phospho-AKT (Ser473) from Cell Signaling Technology (Beverly, MA, USA); monoclonal anti-β-tubulin and anti-GAPDH from Sigma-Aldrich. Signals were detected using appropriate horseradish peroxidase-conjugated secondary antibodies and the ECL system (GE Healthcare, Milan, Italy). Densitometric analysis was performed using Quantity One software (Bio-Rad, Milan, Italy). Densitometry units (U) were calculated relative to GAPDH levels.

### Confocal immunofluorescence microscopy

Cells (2 × 10^5^) were seeded on poly-l-lysine-coated micro cover glasses and fixed with 4% paraformaldehyde for 15 min at room temperature. Cells were then permeabilized with Triton X-100 (0.1% in phosphate-buffered saline (PBS)) for 5 min at room temperature. After three washes in PBS with Triton X-100 0.01%, cells were blocked with blocking buffer (1% bovine serum albumin in PBS) for 40 min before overnight incubation, in a humidified chamber at 4 °C, with the rabbit polyclonal anti-Jag1 C-terminal antibody (HPA021555, dilution 1:200; Sigma-Aldrich), specific for the sequence 1093–1217 of the human Jag1-IC. After three washes, cells were incubated with an Alexa-Fluor 488-conjugated goat anti-rabbit IgG (Thermo Fisher Scientific) for 40 min in the dark. Nuclei were stained with 4,6-DiAmidino-2- Phenyl Indole (DAPI) in ProLong Gold antifade mounting reagent (Thermo Fisher Scientific). Images were acquired with a laser scanning confocal microscope LSM 800 with Airyscan (Zeiss, Oberkochen, Germany) using a 63x oil immersion and 1.4 NA objective.

### Quantitative real-time PCR

RNA was extracted using RNeasy Plus Kit (Qiagen, Hilden, Germany), and cDNA was obtained using Prime Script RT Master Mix (Takara Bio, Dalian, China). Real-time quantitative PCR was performed with PCR Master Mix Power SYBR Green (Applied Biosystems, Warrington, UK), using the 7900HT Fast Real-Time PCR System (Applied Biosystems). The sequences of primers used for *JAG1*, *NOTCH1*, *NOTCH2* and *GAPDH* analysis are shown in Supplementary Table S3. The expression of each target gene was normalized to *GAPDH*, and relative fold change was calculated using the 2^−^^ΔΔCt^ method.

### siRNA transfection

CLL cells were transfected using the Amaxa nucleofection technology (Amaxa, Cologne, Germany) and the ON-TARGETplus SMARTpool small interfering RNA (siRNA) to Jag1 (siJag1) or ON-TARGETplus siCONTROL nontargeting pool (siCtrl) as negative control (Dharmacon, Lafayette, CO, USA). CLL cells (12 × 10^6^) were resuspended in 100 µl Cell Line Solution Kit V (Lonza Group Ltd, Basel, Switzerland) with 0.25 μM of siJag1 or siCtrl, transferred to the provided cuvettes and transfected with the Amaxa Nucleofector II device (program U-013). Cells were immediately transferred into 12-well plates in complete medium and cultured for 72 h in the presence of 25 ng/ml IL-4. Cells were then examined for Jag1 protein expression to verify the efficiency of silencing, and for cell viability/apoptosis.

### Statistical analyses

Statistical analyses were performed using GraphPad Prism 5 software (GraphPad Software, Inc., La Jolla, CA). The data are presented as mean ± SD. Statistical differences between mean values were evaluated using the Student’s *t* test. Jag1-FL expression in different prognostic groups was compared using the Mann-Whitney test. The OS, defined as time from diagnosis to death, was estimated by the Kaplan–Meier method, and the log-rank test was used to compare differences between survival curves. Results were considered statistically significant with *P* value < 0.05 (^*^P < 0.05, ^**^P < 0.01, ^***^P < 0.001).

## Electronic supplementary material

Supplementary Information
