# Supplementary material for: IL-4-dependent Jagged1 expression/processing is associated with survival of chronic lymphocytic leukemia cells but not with Notch activation
Source: Cell Death Dis. 2018 Nov 26;9(12):1160. doi: 10.1038/s41419-018-1185-6 (PMC6255763; doi:10.1038/s41419-018-1185-6)
Supplement: Supplementary file 1 — Supplementary Information [file 41419_2018_1185_MOESM1_ESM.pdf]

SUPPLEMENTARY INFORMATION

IL-4-dependent Jagged1 expression/processing is associated with survival of chronic lymphocytic leukemia cells but not with Notch activation

Filomena De Falco[1], Beatrice Del Papa[1], Stefano Baldoni[2], Rita Sabatini[1], Franca Falzetti[1], Mauro Di Ianni[3,4], Maria Paola Martelli[1], Federica Mezzasoma[1], Maria Pelullo[5], Pierfrancesco Marconi[6], Paolo Sportoletti[1], Isabella Screpanti[5] and Emanuela Rosati[6]

[1]Department of Medicine, Institute of Hematology - Centro di Ricerche Emato-Oncologiche (CREO), University of Perugia, Perugia, Italy

[2]Department of Life, Health and Environmental Sciences, Hematology Section, University of L'Aquila, L'Aquila, Italy

[3]Department of Medicine and Aging Sciences, University of Chieti-Pescara, Chieti, Italy

[4]Department of Hematology, Transfusion Medicine and Biotechnologies, Ospedale Civile, Pescara, Italy

[5]Department of Molecular Medicine, University of Rome "La Sapienza", Rome, Italy

[6]Department of Experimental Medicine, Biosciences and Medical Embryology Section, University of Perugia, Perugia, Italy

Contents:

List of Supplementary Tables:

Table S1: Characteristics of CLL patients

Table S2: Expression levels of Jag1-FL in CLL samples

Table S3: Primers used for quantitative real-time PCR

List of Supplementary Figures:

Figure S1**:** Full images of the cropped blots shown in Figure 1a-c

Figure S2: Full images of the cropped blots shown in Figure 2a, b

Figure S3: The expression of Jag1-IC is absent in normal PBL

Figure S4: Full images of the cropped blots shown in Figure 4a, c

Figure S5: Full images of the cropped blots shown in Figure 5a, b

Figure S6: Full images of the cropped blots shown in Figure 6a

Figure S7: Full images of the cropped blots shown in Figure 7a, c

Figure S8: Full images of the cropped blots shown in Figure 8a

Figure S9: Full images of the cropped blots shown in Figures 9a, c

Supplementary Table S1: Characteristics of CLL patients

**Patients**	**Binet stage**	***IgV$_H$* status[1]**	**ZAP70 expression[2]**	**CD38 expression[3]**	***NOTCH1* status (% mutant allele burden)[4]**	**Cytogenetics**
**CLL1**	A	Unm	+	+	Unm	del 11
**CLL2**	B	Unm	-	-	Unm	ND
**CLL3**	A	Mut	+	-	Unm	ND
**CLL4**	A	Unm	+	+	Unm	del 11<br>del 13
**CLL5**	B	Mut	+	-	Unm	Normal
**CLL6**	A	Mut	+	-	Unm	Normal
**CLL7**	A	Mut	-	-	Unm	del 13
**CLL8**	B	Unm	+	-	Mut<br>(1.9)	Normal
**CLL9**	A	Unm	-	+	Unm	ND
**CLL10**	B	Mut	-	-	Unm	Normal
**CLL11**	A	Unm	+	-	Unm	ND
**CLL12**	A	Unm	+	-	Unm	del 11<br>del 13
**CLL13**	C	Unm	-	-	Mut<br>(1.3)	del 11<br>del 13<br>del 14
**CLL14**	B	Mut	+	-	Unm	ND
**CLL15**	C	Unm	-	-	Unm	del 14
**CLL16**	B	Mut	-	-	Unm	ND
**CLL17**	A	Mut	+	-	Unm	Normal
**CLL18**	A	Mut	-	-	Unm	Normal
**CLL19**	A	Mut	+	+	Unm	ND
**CLL20**	B	Unm	-	-	Unm	Normal
**CLL21**	A	Mut	-	-	Unm	Normal

Abbreviations: Mut, mutated; Unm, unmutated; ND, not determined.
[1]Mutated was defined as having a frequency of mutations >2% from germline *VH*.
[2]Positivity refers to detection of >20% $ZAP70^+/CD19^+$.
[3]Positivity refers to detection of >20% $CD38^+/CD19^+$.
[4]c.7544_7545delCT in *NOTCH1* exon 34.
[5]Assessed by FISH.

Supplementary Table S2: Expression levels of Jag1-FL in CLL samples

Patients	Jag1-FL levels[1]
**CLL1**	0.55
**CLL2**	0.57
**CLL3**	0.50
**CLL4**	0.53
**CLL5**	0.37
**CLL6**	0.72
**CLL7**	0.71
**CLL8**	0.44
**CLL9**	0.24
**CLL10**	0.71
**CLL11**	0.39
**CLL12**	0.17
**CLL13**	0.68
**CLL14**	0.54
**CLL15**	0.33
**CLL16**	0.82
**CLL17**	1.07
**CLL18**	0.36
**CLL19**	0.20
**CLL20**	0.33
**CLL21**	0.92

[1]Jag1-FL levels were measured as a ratio of Jag1-FL to GAPDH levels, evaluated by Western blot analysis using the Jag1 C-20 antibody.

Supplementary Table S3: Primers used for quantitative real-time PCR

**Gene symbol**	**Forward primer (5’ to 3’)**	**Reverse primer (5’ to 3’)**
JAG1	TGCCAGGAAGTTTCAGGGAGA	TTGGCCCCATCTGGTATCACA
NOTCH1	GAGGCGTGGCAGACTATGC	CTTGTACTCCGTCAGCGTGA
NOTCH2	TATTGATGACTGCCCTAACCACA	ATAGCCTCCATTGCGGTTGG
GAPDH	ATGGGGAAGGTGAAGGTCG	GGGGTCATTGATGGCAACAATA

Supplementary Figure S1

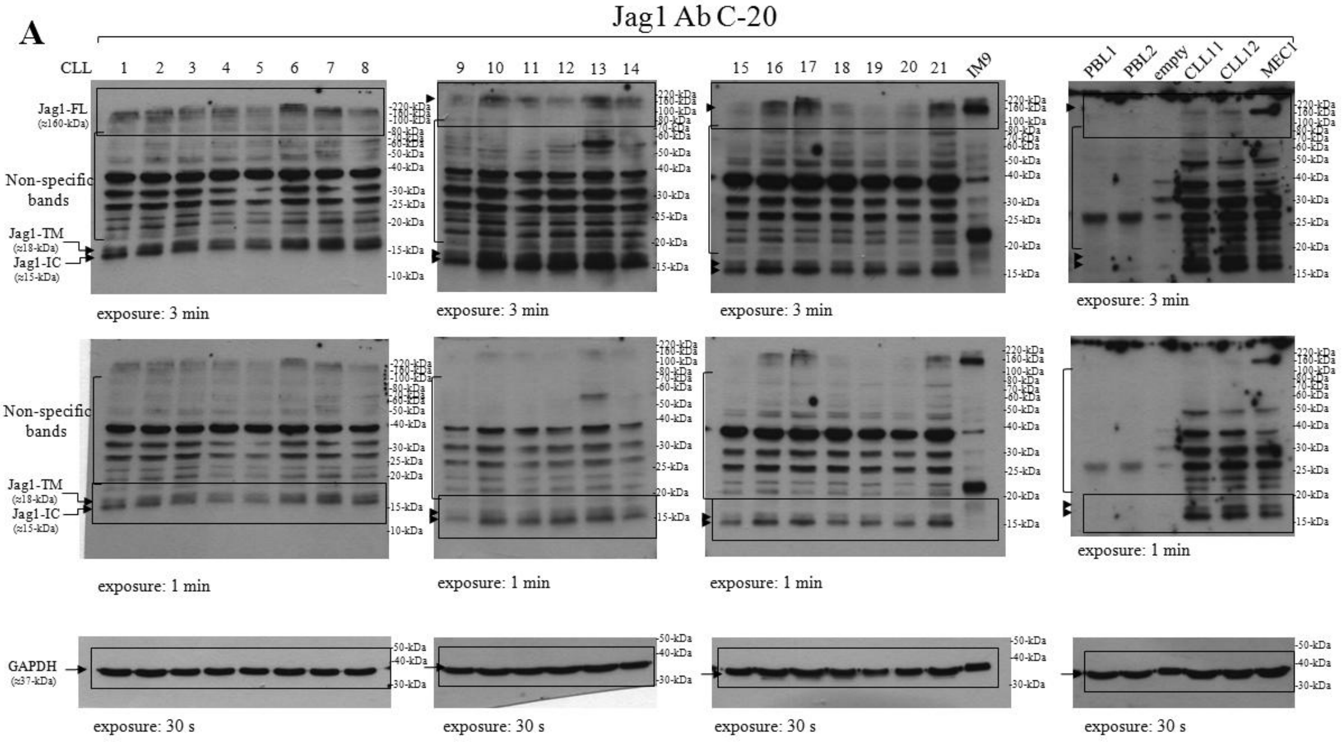

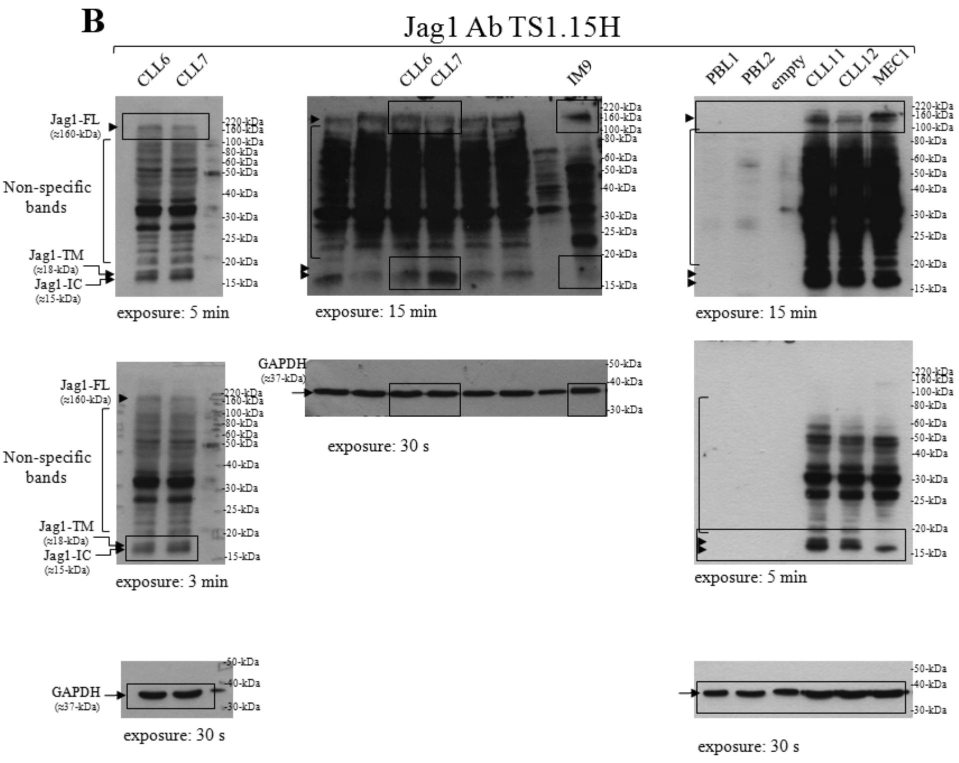

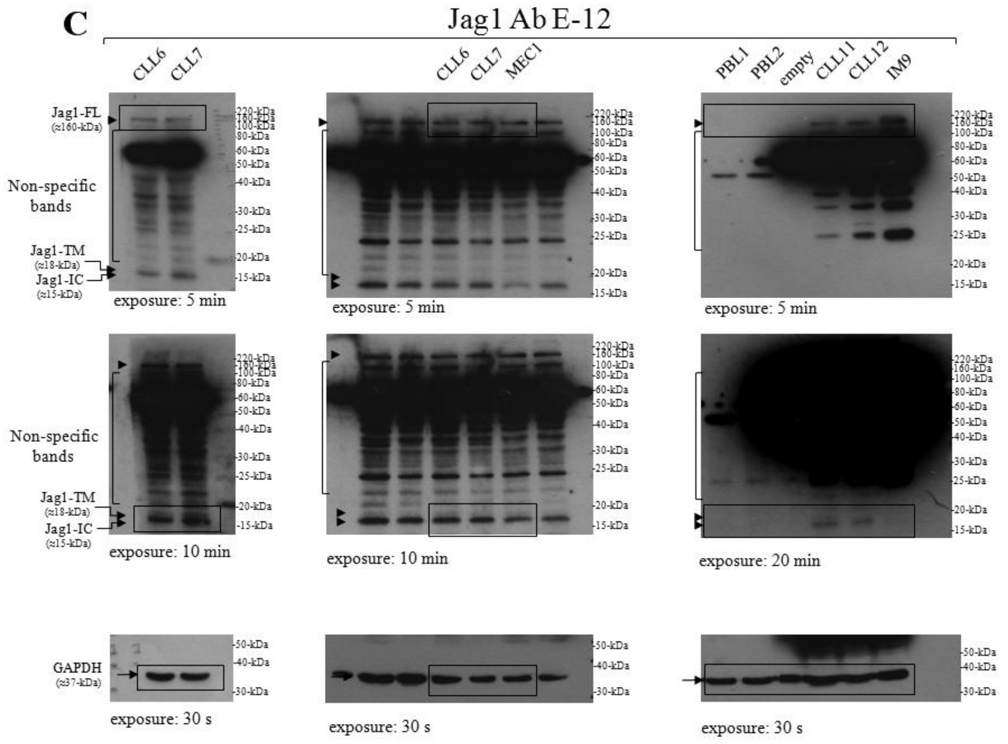

Supplementary Figure S1. Full images of the cropped blots shown in Figure 1. (A) Full images of the cropped blots shown in Figure 1a. The membranes were first hybridized with the Jag1 C-20 antibody (Ab) and then, after stripping, with an anti-GAPDH Ab. For each Jag1 blot, two different exposure times are shown. The areas of immunoreactive Jag1-FL, Jag1-TM, Jag1-IC and GAPDH, shown in Figure 1a, are within black boxes. Molecular weight standards are shown on the right of each blot. In the blot with PBL, an empty lane has been placed between PBL2 and CLL11 to avoid cross-contamination. **(B) Full images of the cropped blots shown in Figure 1b.** The membranes were first hybridized with the Jag1 TS1.15H Ab and then, after stripping, with an anti-GAPDH Ab. For Jag1 blots on the right and on the left, two different exposure times are shown. The areas of immunoreactive Jag1-FL, Jag1-TM, Jag1-IC and GAPDH, shown in Figure 1b, are within black boxes.

Molecular weight standards are shown on the right of each blot. In the blot with PBL, an empty lane has been placed between PBL2 and CLL11 to avoid cross-contamination. **(C) Full images of the cropped blots shown in Figure 1c.** The membranes were first hybridized with the Jag1 E-12 Ab and then, after stripping, with an anti-GAPDH Ab. For each Jag1 blot, two different exposure times are shown. Areas of immunoreactive Jag1-FL, Jag1-TM, Jag1-IC and GAPDH, shown in Figure 1c, are within black boxes. Molecular weight standards are shown on the right of each blot. In the blot with PBL, an empty lane has been placed between PBL2 and CLL11 to avoid cross-contamination.

Supplementary Figure S2

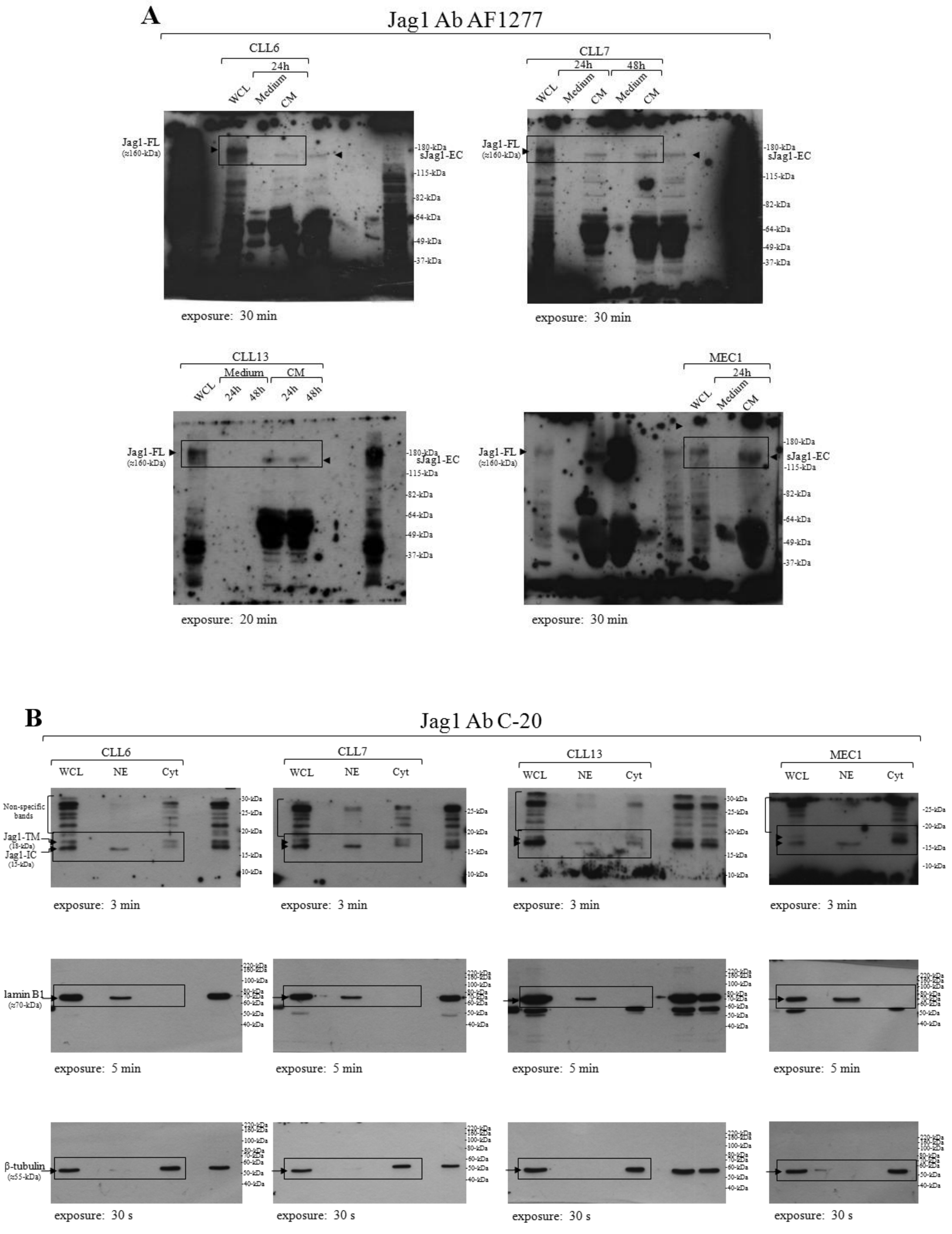

Supplementary Figure S2. Full images of the cropped blots shown in Figure 2a,b. (A) Full images of the cropped blots shown in Figure 2a. The membranes were hybridized with the N-terminal Jag1 AF1277 antibody. The areas of immunoreactive Jag1-FL and sJag1-EC, shown in Figure 2a, are within black boxes. Molecular weight standards are shown on the right of each blot. **(B) Full images of the cropped blots shown in Figure 2b.** The membranes were cut into two parts at level of about 30-kDa. The membranes with proteins larger than 30-kDa were first hybridized with an anti-β-tubulin antibody and then, after stripping, with an anti-lamin B1 antibody. The membranes with proteins smaller than 30-kDa were hybridized with the anti-Jag1 C-20 antibody. The areas of immunoreactive Jag1-TM, Jag1-IC, lamin B1 and β-tubulin, shown in Figure 2b, are within black boxes. Molecular weight standards are shown on the right of each blot.

Supplementary Figure S3

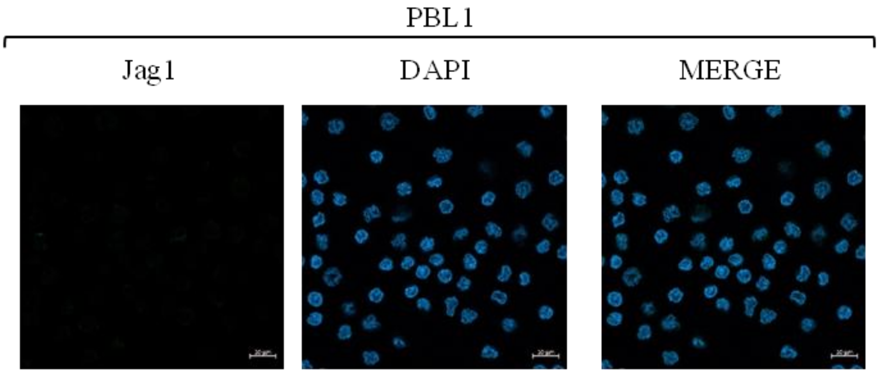

Supplementary Figure S3. The expression of Jag1-IC is absent in normal PBL. Confocal microscopy images of a representative PBL sample (n=3) stained with the C-terminal Jag1 HPA021555 antibody (green) and with DAPI for nuclei (blue), as described in “Materials and methods”. Scale bars: 10 µm.

Supplementary Figure S4

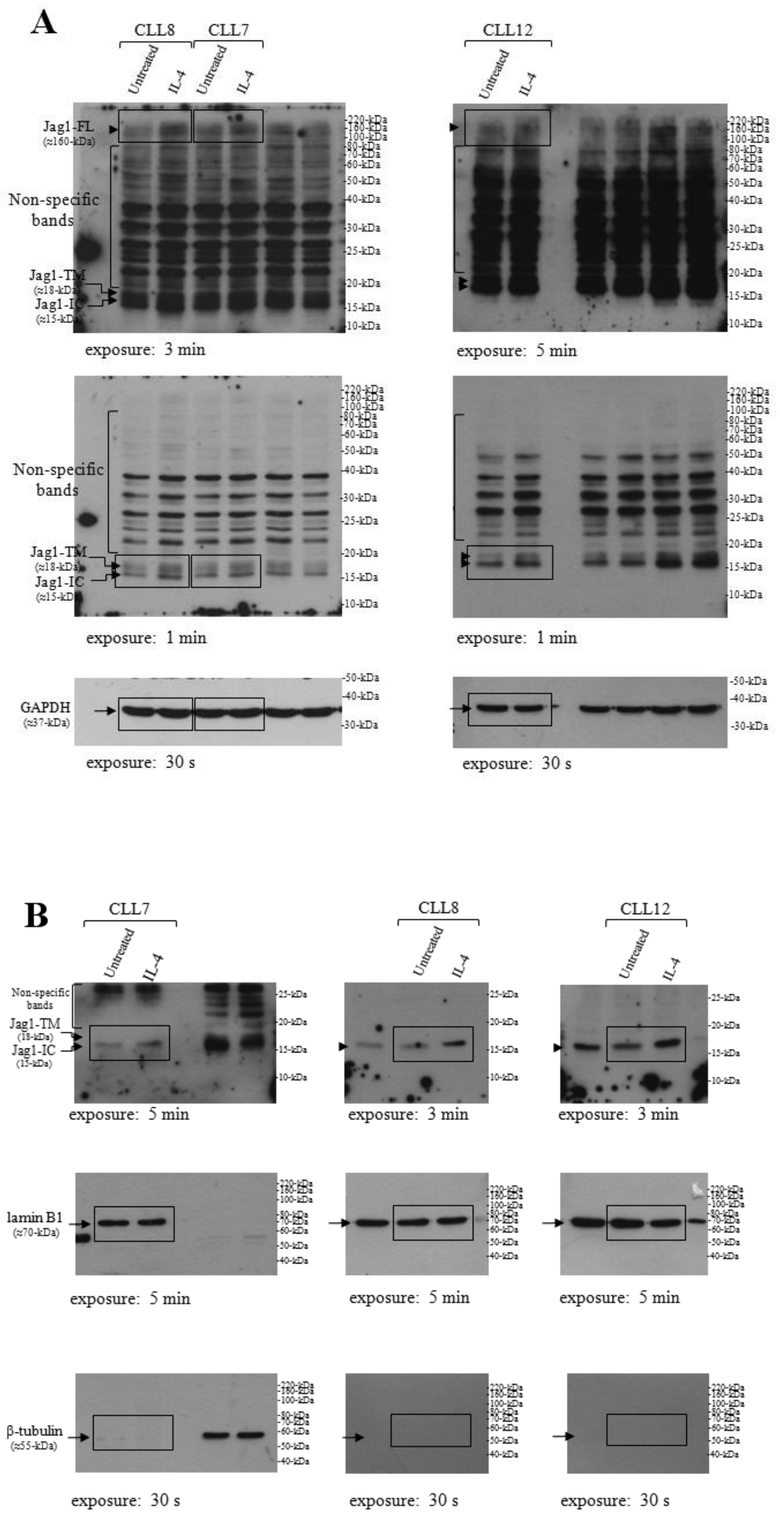

Supplementary Figure S4. Full images of the cropped blots shown in Figure 4a,c.

(A) Full images of the cropped blots shown in Figure 4a. The membranes were first hybridized with the Jag1 C-20 antibody and then, after stripping, with an anti-

GAPDH antibody. For each blot, two different exposure times are shown. The areas of immunoreactive Jag1-FL, Jag1-TM, Jag1-IC and GAPDH, shown in Figure 4a, are within black boxes. Molecular weight standards are shown on the right of the blots. **(B) Full images of the cropped blots shown in Figure 4c.** The membranes were cut into two parts at level of about 30-kDa. The membranes with proteins larger than 30-kDa were first hybridized with an anti-β-tubulin antibody and then, after stripping, with an anti-lamin B1 antibody. The membranes with proteins smaller than 30-kDa were hybridized with the Jag1 C-20 antibody. The areas of immunoreactive Jag1-IC, lamin B1 and β-tubulin, shown in Figure 4c, are within black boxes. Molecular weight standards are shown on the right of each blot.

Supplementary Figure S5

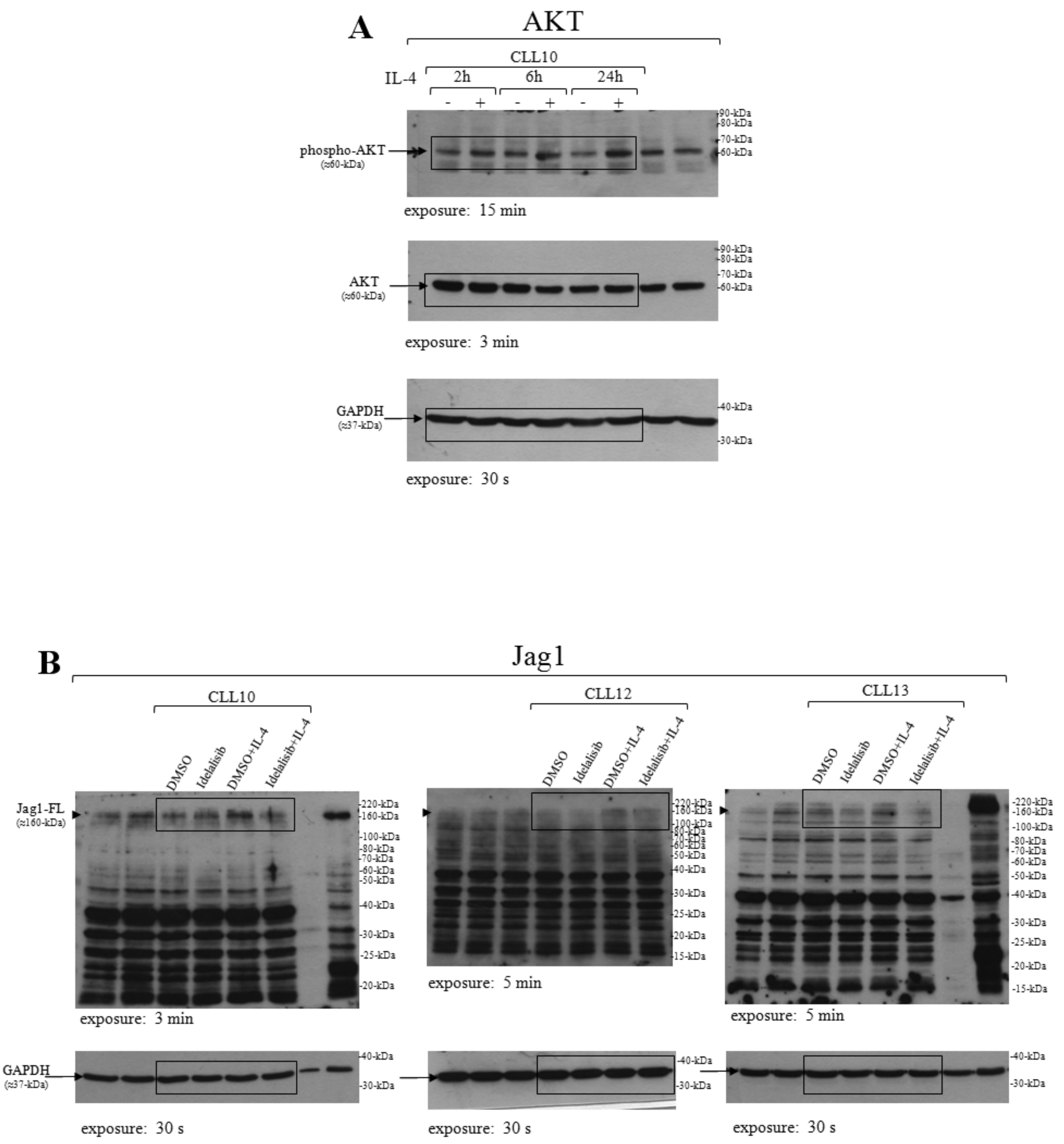

Supplementary Figure S5. Full images of the cropped blots shown in Figure 5a,b. (A) Full images of the cropped blot shown in Figure 5a. The membrane was cut into two parts at level of about 50-kDa. The membrane with proteins larger than 50-kDa was first hybridized with an anti-phospho-AKT antibody and then, after stripping, with an anti-total AKT antibody. The membrane with proteins smaller than

50-kDa was hybridized with an anti-GAPDH antibody. The areas of immunoreactive phospho-AKT, total AKT and GAPDH, shown in Figure 5a, are within black boxes. Molecular weight standards are shown on the right of the blot. **(B) Full images of the cropped blots shown in Figure 5b.** The membranes were first hybridized with the Jag1 C-20 antibody and then, after stripping, with an anti-GAPDH antibody. In each blot, the areas of immunoreactive Jag1-FL and GAPDH, shown in Figure 5b, are within black boxes. Molecular weight standards are shown on the right of each blot.

Supplementary Figure S6

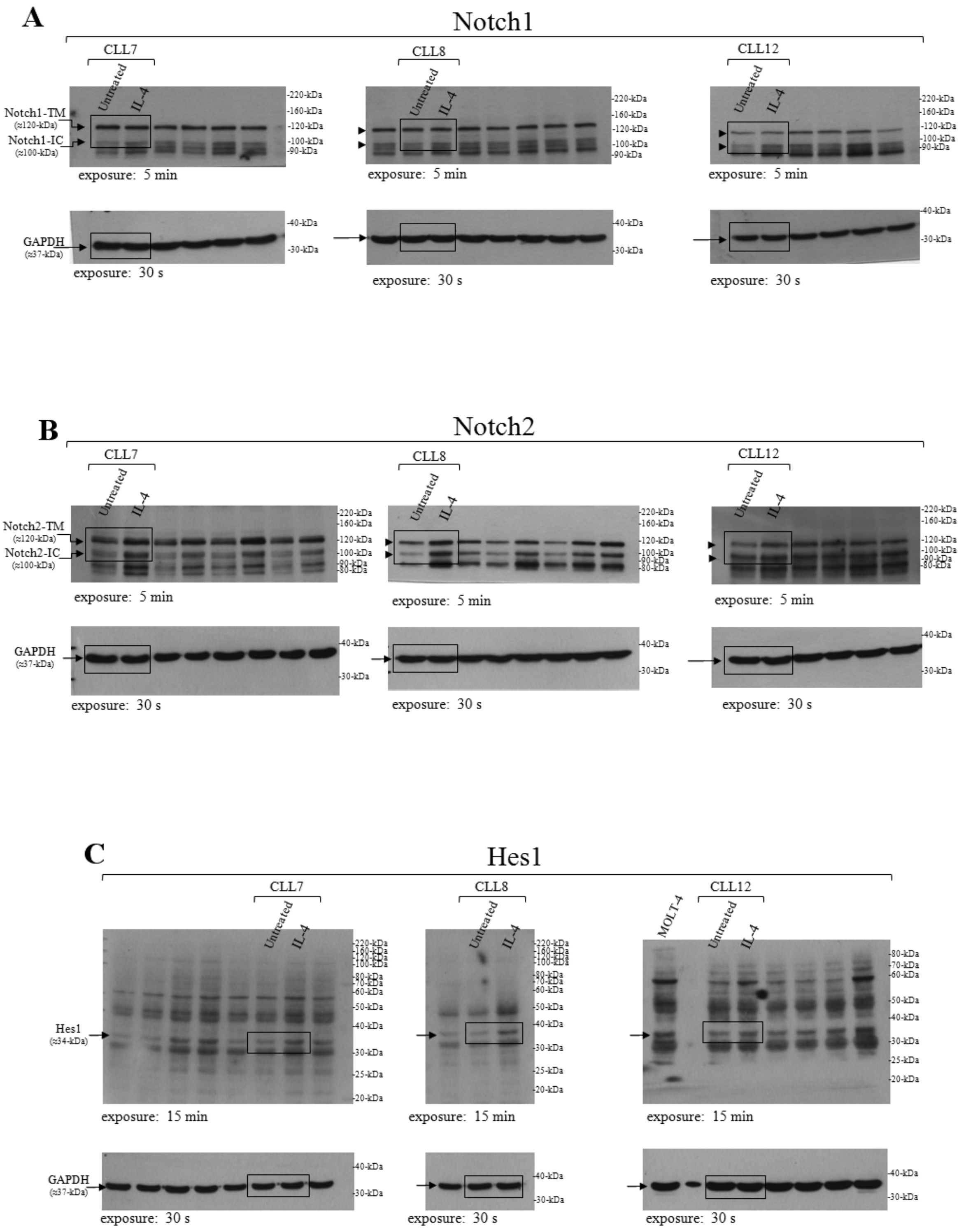

Supplementary Figure S6. Full images of the cropped blots shown in Figure 6a.

The membranes analyzed for Notch1 (**A**) and Notch2 (**B**) were cut into two parts at

level of about 80-kDa. The membranes with proteins larger than 80-kDa were hybridized with an anti-Notch1 (**A**) or an anti-Notch2 (**B**) antibody. The membranes with proteins smaller than 80-kDa were hybridized with an anti-GAPDH antibody. In each blot, the areas of immunoreactive Notch1 (TM and IC), Notch2 (TM and IC) and of the respective GAPDH, shown in Figure 6a, are within black boxes. Molecular weight standards are shown on the right of each blot. (**C**) The membranes were first hybridized with an anti-Hes1 antibody and then, after stripping, with an anti-GAPDH antibody. In CLL12 blot, whole-cell lysates from MOLT-4 cell line were used as a positive control for Hes1 expression. This lane is not included in the blot shown in Figure 6a. In each blot, the areas of immunoreactive Hes1 and GAPDH, shown in Figure 6a, are within black boxes. Molecular weight standards are shown on the right of each blot.

Supplementary Figure S7

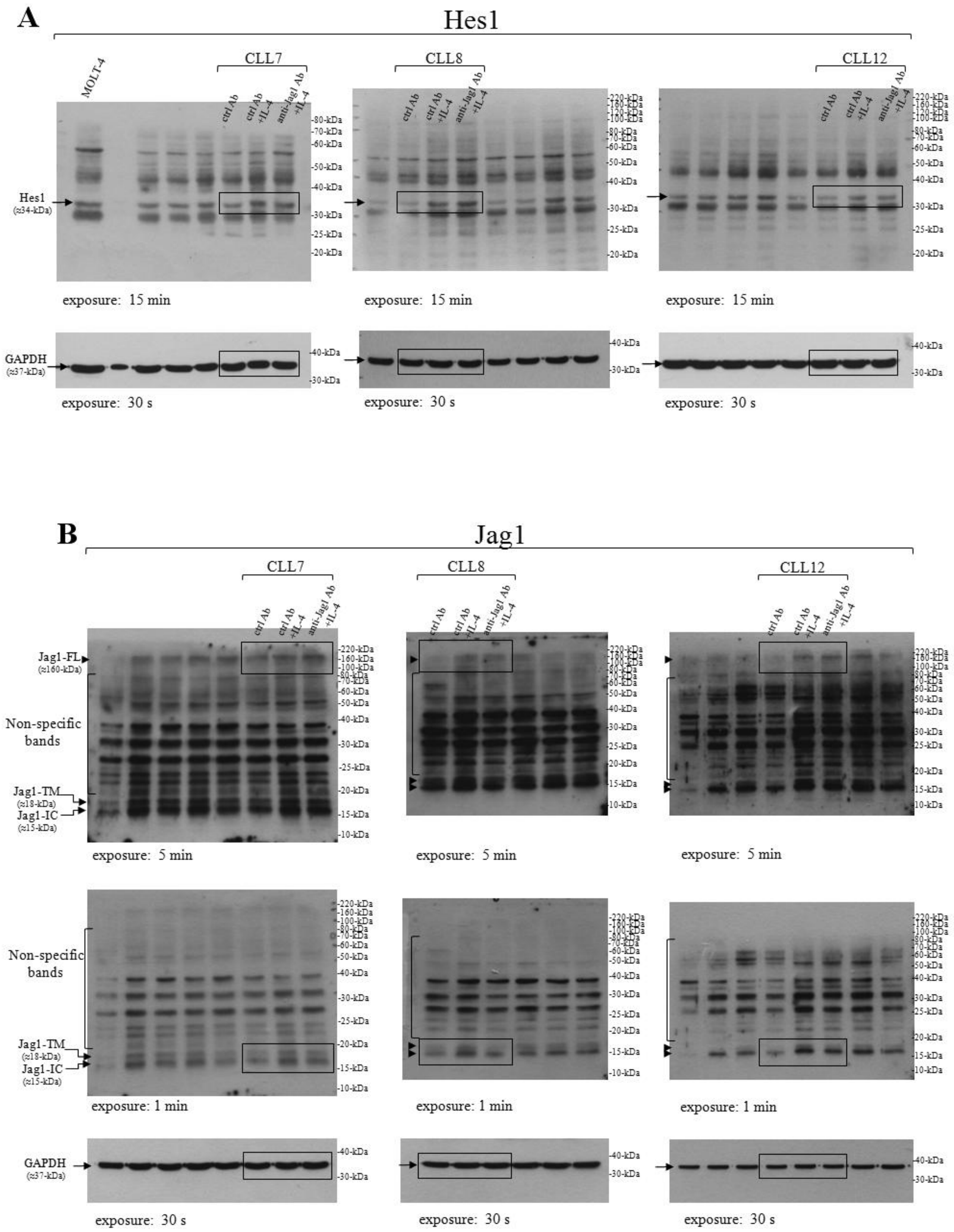

Supplementary Figure S7. Full images of the cropped blots shown in Figure 7a,c.

(A) Full images of the cropped blots shown in Figure 7a. The membranes were first hybridized with an anti-Hes1 antibody and then, after stripping, with an anti-

GAPDH antibody. In CLL7 blot, whole-cell lysates from MOLT-4 cell line were used as a positive control for Hes1 expression. This lane is not included in the blot shown in Figure 7a. In each blot, the areas of immunoreactive Hes1 and GAPDH, shown in Figure 7a, are within black boxes. Molecular weight standards are shown on the right of each blot. **(B) Full images of the cropped blots shown in Figure 7c.** The membranes were first hybridized with the Jag1 C-20 antibody and then, after stripping, with an anti-GAPDH antibody. For each blot, two different exposure times are shown. In each blot, the areas of immunoreactive Jag1-FL, Jag1-TM, Jag1-IC and GAPDH, shown in Figure 7c, are within black boxes. Molecular weight standards are shown on the right of each blot.

Supplementary Figure S8

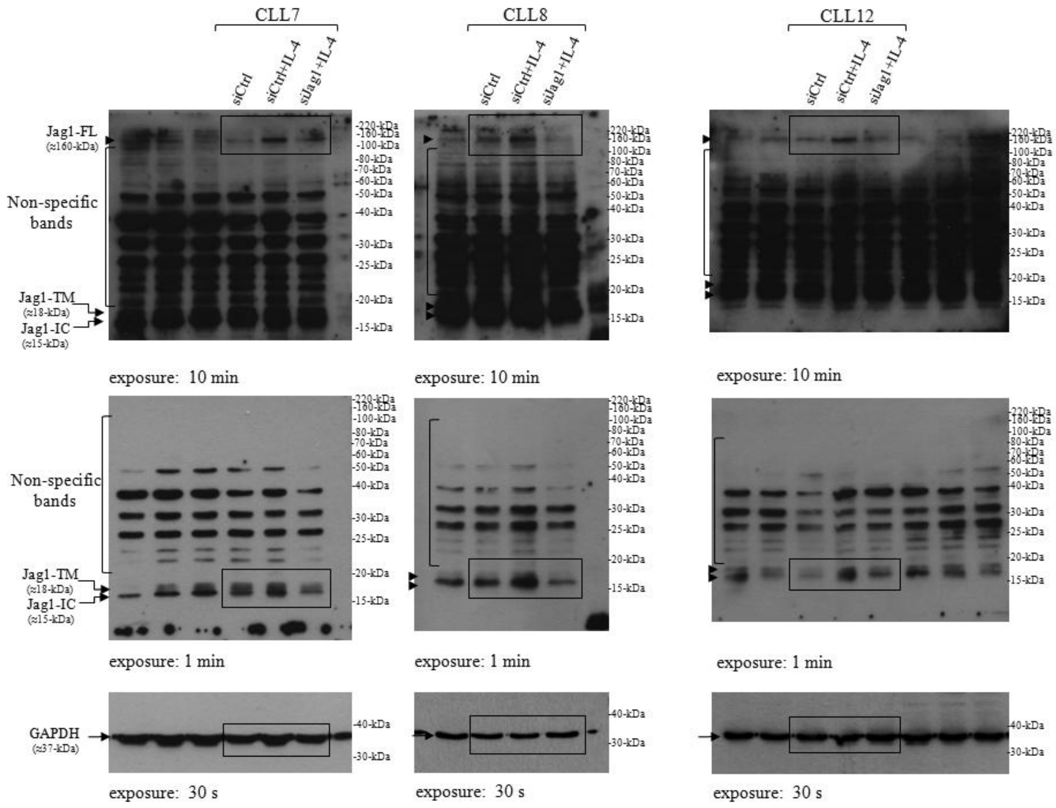

Supplementary Figure S8. Full images of the cropped blots shown in Figure 8a. The membranes were first hybridized with the Jag1 C-20 antibody and then, after stripping, with an anti-GAPDH antibody. For each blot, two different exposure times are shown. The areas of immunoreactive Jag1-FL, Jag1-TM, Jag1-IC and GAPDH, shown in Figure 8a, are within black boxes. Molecular weight standards are shown on the right of each blot.

Supplementary Figure S9

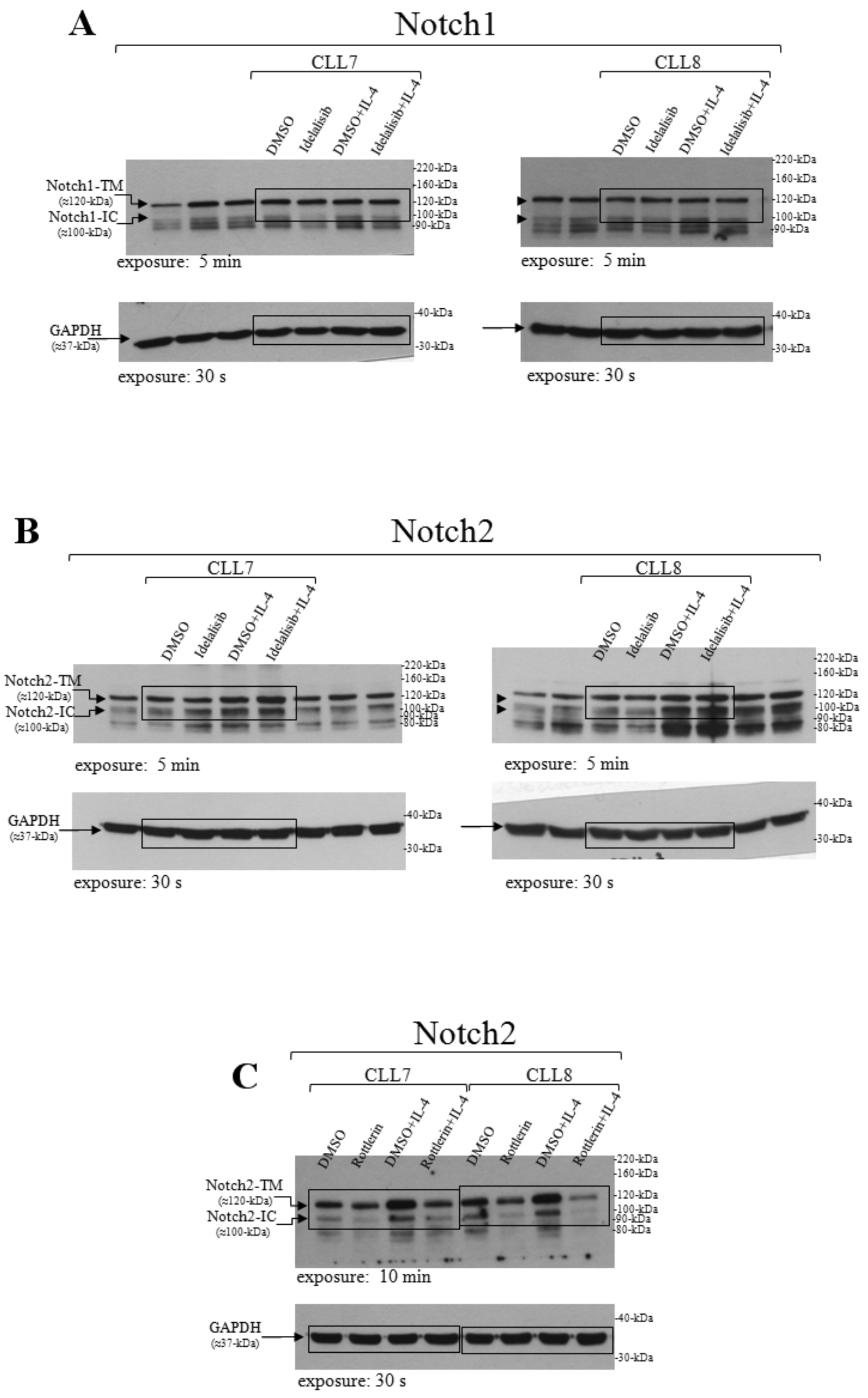

Supplementary Figure S9. Full images of the cropped blots shown in Figure 9a,c.

Full images of the cropped blots shown in Figure 9a. The membranes analyzed for

Notch1 **(A)** and Notch2 **(B)** were cut into two parts at level of about 80-kDa. The membranes with proteins larger than 80-kDa were hybridized with an anti-Notch1 **(A)** or an anti-Notch2 **(B)** antibody. The membranes with proteins smaller than 80-kDa were hybridized with an anti-GAPDH antibody. In each blot, the areas of immunoreactive Notch1 (TM and IC), Notch2 (TM and IC) and GAPDH, shown in Figure 9a, are within black boxes. Molecular weight standards are shown on the right of each blot. **(C) Full images of the cropped blots shown in Figure 9c.** The membranes were cut into two parts at level of about 80-kDa. The membranes with proteins larger than 80-kDa were hybridized with an anti-Notch2 antibody. The membranes with proteins smaller than 80-kDa were hybridized with an anti-GAPDH antibody. In each blot, the areas of immunoreactive Notch2 (TM and IC) and GAPDH, shown in Figure 9c, are within black boxes. Molecular weight standards are shown on the right of each blot.
